# *Drosophila* Ovarian Germline Stem Cell Cytocensor Projections Dynamically Receive and Attenuate BMP Signaling

**DOI:** 10.1016/j.devcel.2019.05.020

**Published:** 2019-08-05

**Authors:** Scott G. Wilcockson, Hilary L. Ashe

**Affiliations:** 1 Faculty of Biology, Medicine and Health, University of Manchester, Manchester M13 9PT, UK

**Keywords:** BMP signaling, *Drosophila*, germline stem cell, cytoskeleton, niche, Dpp, Tkv, signaling filopodia

## Abstract

In the *Drosophila* ovarian germline, Bone Morphogenetic Protein (BMP) signals released by niche cells promote germline stem cell (GSC) maintenance. Although BMP signaling is known to repress expression of a key differentiation factor, it remains unclear whether BMP-responsive transcription also contributes positively to GSC identity. Here, we identify the GSC transcriptome using RNA sequencing (RNA-seq), including the BMP-induced transcriptional network. Based on these data, we provide evidence that GSCs form two types of cellular projections. Genetic manipulation and live *ex vivo* imaging reveal that both classes of projection allow GSCs to access a reservoir of Dpp held away from the GSC-niche interface. Moreover, microtubule-rich projections, termed “cytocensors”, form downstream of BMP and have additional functionality, which is to attenuate BMP signaling. In this way, cytocensors allow dynamic modulation of signal transduction to facilitate differentiation following GSC division. This ability of cytocensors to attenuate the signaling response expands the repertoire of functions associated with signaling projections.

## Highlights

• Identification of the transcriptomes in GSCs and their differentiating daughters
• Dpp is sequestered by Dally at the anterior face of niche cells, away from GSCs
• GSCs synthesize two types of cellular projections to access and receive Dpp
• Dpp-induced cytocensor projections also attenuate Dpp signaling to tune the response

## Graphical Abstract

**Figure:**
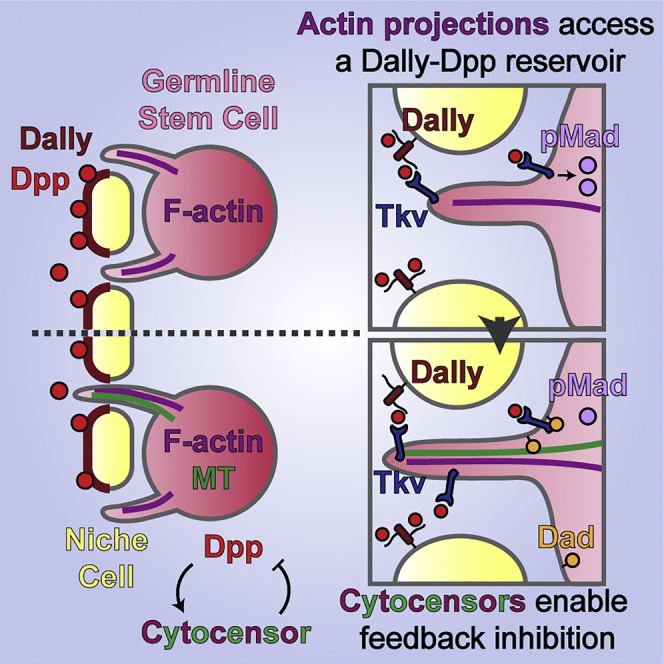

## Teaser

By identifying the *Drosophila* ovarian germline stem cell (GSC) transcriptome, Wilcockson and Ashe show that GSCs synthesize two classes of cellular projections to receive the self-renewal BMP signal. One class, the BMP-induced microtubule-based “cytocensor” projection, also attenuates BMP signaling in GSCs, allowing them to calibrate their signal response.

## Introduction

The stem cell niche is a tissue microenvironment, specialized in structure and function, that ensures the self-renewal and survival of cells needed to maintain tissue homeostasis throughout an organism’s life. The first niche was characterized in the *Drosophila* ovarian germline (Cox et al., 1998, King and Lin, 1999) where the Bone Morphogenetic Protein (BMP) family member, Decapentaplegic (Dpp), was found to be necessary for maintenance of germline stem cells (GSCs) (Xie and Spradling, 1998, Xie and Spradling, 2000). Since this discovery, there has been an explosion in the identification and characterization of stem cell niches in most tissues and model organisms (Scadden, 2014).

Within the *Drosophila* ovary, GSCs are maintained at the anterior tip in discrete structures called germaria (Lin and Spradling, 1993). A small population of somatic cells, the cap cells (CpCs), contact the GSCs through E-cadherin (Ecad)-based adherens junctions (AJs) (Song et al., 2002) and promote stem cell identity through the secretion of Dpp homodimers or Dpp-Glassbottom boat (Gbb) heterodimers. Dpp signals at an exquisitely short range to maintain 2–3 GSCs per niche. Upon cell division, one daughter cell exits the niche, allowing it to move out of the range of the Dpp signal and differentiate into a cystoblast (CB). Multiple mechanisms have been described for restricting Dpp range, including stabilization or concentration of Dpp within the niche by the heparan sulphate proteoglycan (HSPG) Divisions abnormally delayed (Dally), sequestration by a collagen IV (CollIV) matrix between the GSCs and CpCs, and escort cell (EC) expression of the Dpp receptor, Thickveins (Tkv), which acts as a “decoy” to soak up any free BMP ligand (Wilcockson et al., 2017). The most anterior ECs thus define the posterior limit of the GSC niche and contact the differentiating CBs to create a differentiation niche.

Within GSCs, the BMP signal is transduced by phosphorylation and activation of the Smad1/5 ortholog, Mothers against Dpp (Mad). Mad oligomerizes with the Smad4 ortholog Medea, leading to their nuclear accumulation (Hamaratoglu et al., 2014). A key Dpp target gene in GSCs is *bag of marbles* (*bam*), encoding an essential differentiation factor, which is repressed by Dpp signaling (Song et al., 2004, Chen and McKearin, 2003). Upon cell division, the daughter cell that exits the niche derepresses *bam*, which initiates the differentiation program. However, few Dpp target genes have been identified in GSCs, and there is little understanding of how the BMP self-renewal signal may positively act on GSC identity. Therefore, we used RNA sequencing (RNA-seq) to identify the GSC transcriptional network, including genes that are regulated by the Dpp signal. These data reveal that the GSC synthesizes different types of cellular projections that function to receive the niche BMP signal, including one class that also plays an active role in BMP signal attenuation, which we thus refer to as “cytocensors.”

## Results

### RNA-Seq of GSC-like Cells and CBs Reveals Putative GSC Self-Renewal and Maintenance Factors

**Figure 1:**
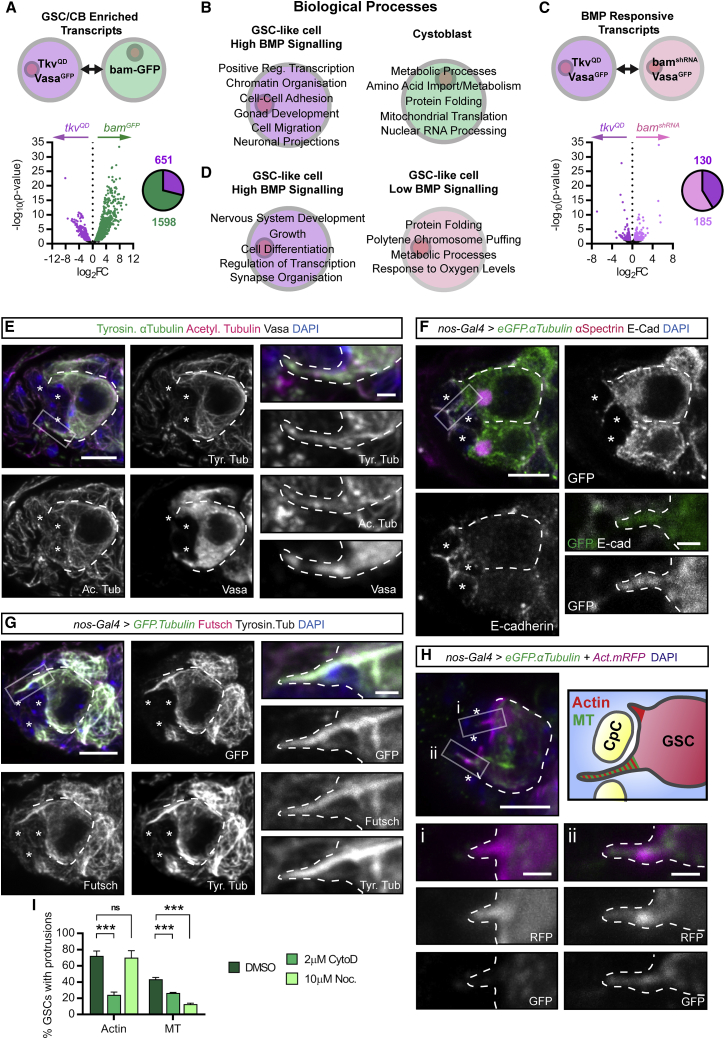
GSCs Upregulate MT-Associated Factors and Extend Cytoskeletal Projections into the Niche (A) Differential expression analysis of RNA enriched in *tkv*^*QD*^ (magenta) and *bam.GFP* (green) expressing GSC-like cells and CBs, respectively. Pie chart shows number of significantly enriched genes for each cell type (log_2_-fold change >0.5, p < 0.05). (B) GO term analysis results showing biological processes enriched in *tkv*^*QD*^ (magenta) and *bam.GFP* (green)-expressing cells. (C and D) as in (A) and (B), showing differential expression (C) and GO term analysis results (D) comparing *tkv*^*QD*^ (magenta) and *bam*^*KD*^ (light pink) expressing GSC-like cells. (E) The MT network of the germarium. GSCs are marked by Vasa expression (white). MTs are labeled by the MT markers, acetylated α-tubulin and tyrosinated α-tubulin. (Inset) Close-up views of the indicated boxed region showing a GSC MT-rich cytoplasmic projection. (F and G) Immunofluorescence staining of germaria with germline *eGFP.αTub* expression. GSCs marked by the spectrosome visualized with anti-αSpectrin, Ecad outlines the CpCs (^∗^) in (F). (G) The MT-associated factor Futsch also localizes to GSC MT-rich projections. (Inset) Close-up views of the indicated boxed regions. (H) GSCs form different actin-rich projections. Immunofluorescence staining of germaria with germline expression of *eGFP.αTub* and *Act*.*mRFP*. A GSC extends one MT- and actin-rich projection (i) and one actin-based filopodium (ii). (I) Percentage of GSCs forming projections after 30 min *ex vivo* treatment of *nos-Gal4>Act42A.GFP* or *eGFP.αTub* ovaries with DMSO (control), 2 μM CytoD, or 10 μM nocodazole. Mean and SD from n > 100 GSCs; 3 biological replicates; ns, not significant. Dashed lines outline individual GSCs and (insets) projections. (^∗^) CpCs. Scale bar, 5 μm or 1 μm (insets). ^∗∗∗^p < 0.0001. See also Figures S1 and S2.

In order to identify regulators of GSC self-renewal and differentiation, we compared the GSC and CB transcriptomes by purifying these cells based on the expression of known cellular markers. Expression patterns and further information on the cell types and signaling circuitry in the germarium are shown in Figures S1A–S1F. In the absence of a GSC-specific marker, we genetically expanded the GSC population by expressing constitutively active Tkv (*UASp-tkv*^*QD*^) using a maternal germline *Gal4* driver (*nos-Gal4::VP16*) in a *vasa*^*GFP*^ background. Vasa is a germ cell marker that we used to isolate GSCs by fluorescence-activated cell sorting (FACS) (Figure 1A; Sano et al., 2002). Flies of this genotype form tumors of pMad^+^ GSC-like cells identifiable by a single, round spectrosome (Figure S1G), a germline-specific spectrin-rich endomembrane organelle that becomes branched in more developed cysts. CBs were isolated by FACS based on their expression of a *bam*-*GFP* reporter and as single cells to exclude more developed GFP^+^ cysts (Figure S1E; Chen and McKearin, 2003). Differential expression analysis revealed 2,249 differentially expressed genes with around one-third up-regulated in *tkv*^*QD*^ (GSCs) and two-thirds up-regulated in *bam-GFP*-expressing cells (CBs) (Figure 1A; Table S1), including *bam*. Gene Ontology (GO) term analysis of GSC- and CB-enriched transcripts reveals distinct biological processes (Figure 1B), including nervous system development and cell migration for GSCs. Enriched transcripts within these categories encode adhesion proteins, axon guidance molecules, ciliogenesis factors, and structural and/or cytoskeletal proteins.

Having identified the GSC transcriptome, we next identified the subset of genes specifically regulated by Dpp signaling by using germline-specific RNAi knockdown of *bam* expression (*UASp-bam*^*KD*^), which blocks GSC differentiation. Germ cells that exit the niche continue to divide away from the short-range Dpp signal and therefore form tumors of pMad^−^ GSC-like cells with a single, round spectrosome that can again be isolated through *vasa*^*GFP*^ expression (Figure S1H). Differential expression analysis of *tkv*^*QD*^- and *bam*^*KD*^-expressing GSC-like cells allows the comparison of “high Dpp” and “low Dpp” GSCs, respectively. This reveals around 300 genes differentially regulated by Dpp signaling, with just under half the genes up-regulated in response to Dpp (Figure 1C; Table S2), including *dad* (Casanueva and Ferguson, 2004). GO term analysis identifies processes activated and repressed by Dpp signaling (Figure 1D). Again, genes up-regulated in response to Dpp signaling encode proteins involved in nervous system development and synapse organization, including the master ciliogenesis transcription factor *Rfx* and the microtubule (MT)-associated protein 1B (MAP1B) homolog *futsch*. Together, these data define the early germline transcriptome, from self-renewing GSCs to differentiating daughter CBs, and the subset of this network functioning downstream of Dpp signaling.

### Germline Stem Cells Extend MT- and Actin-Rich Projections into the Niche

Putative Dpp target genes in the GSC transcriptome include *Rfx* and *futsch*, which both regulate the formation of MT-based structures. To investigate a potential cytoskeletal response to Dpp signaling, we first defined the stem cell MT network using immunofluorescence staining of tyrosinated α-tubulin (a marker of new, dynamic MTs), acetylated α-tubulin (a marker of stable MTs), and the germ cell marker Vasa. GSCs are enriched for tyrosinated α-tubulin compared to the post-mitotic CpCs (Figure 1E, CpCs marked by asterisks and the GSC is outlined by a dashed line). This difference enables the visualization of stem cell-derived MT-rich projections that a subset of GSCs extend into the niche (Figure 1E, see box). The cytoplasmic protein, Vasa, also localizes within these projections, confirming that these structures are GSC derived. To further characterize these MT-rich projections, we specifically visualized the stem cell MT network through the germline expression of *UASp*-*eGFP*.*α-Tubulin84B* (*GFP.αTub*). Ecad staining delineates the contact points between individual CpCs and the GSC-niche interface. A subset of GSCs is found to generate a single, short MT-based projection that extends around or between the CpCs (Figures 1F and S2A). These data reveal that ovarian GSCs generate MT-based structures that extend toward and between the niche CpCs.

As our RNA-seq data identified genes associated with ciliogenesis, we addressed whether these MT-rich projections were ciliary in nature. GFP.αTub^+^ projections are composed of acetylated tubulin, a classical ciliary marker (Figures S2B and S2Bi). However, the MTs show a non-uniform pattern of acetylation unlike stable ciliary MTs. In addition, no association of these GSC projections with the centrosome, based on γ-tubulin staining, is observed (Figure S1C). Therefore, we conclude that these MT-rich projections are not ciliary in nature and hereon refer to them as “cytocensors” based on their signal suppression property, i.e., acting as a censor (see Figure 7). GSCs of the *Drosophila* testis generate MT nanotubes whose formation is regulated by ciliary proteins (Inaba et al., 2015). However, our data highlight a number of differences between cytocensors and MT nanotubes, consistent with them being distinct structures (see Discussion).

One of the positive Dpp target genes identified in the RNA-seq is *futsch*. Futsch function is best characterized in the nervous system where it promotes MT stability (Halpain and Dehmelt, 2006). Futsch staining reveals strong GSC enrichment compared with CpCs (Figure 1G). Futsch colocalizes extensively with the stem cell MT network, including the cytocensor MTs. These data are consistent with Dpp signaling up-regulating *futsch* expression and Futsch subsequently localizing to GSC cytocensors where it may play a role in projection stability (see Figure 3).

We next addressed whether these projections contain or require the actin cytoskeleton for their formation by specifically expressing both *GFP*.*αTub* and *UASp-Actin5C.mRFP* in the germline. Immunofluorescence staining reveals that GSCs also generate multiple actin-based projections (APs). Figure 1H shows a single stem cell with two distinct projections: one short actin-based filopodium (Figure 1H, box i) and a longer projection containing both MTs and actin (Figure 1H, box ii). This shows that GSC cytocensors are both MT- and actin-rich projections, while GSCs also generate additional APs.

Having identified that ovarian GSCs extend multiple distinct cellular projections, we investigated the regulatory relationship between actin and MTs in the formation of these projections. We treated *Drosophila* ovaries *ex vivo* with cytochalasin D (CytoD) and nocodazole, inhibitors of actin and MT polymerization, respectively. A short 30-min incubation with CytoD or nocodazole was sufficient to disrupt F-actin (Figure S2D) or reduce tubulin levels (Figure S2E), respectively. Treatment of ovaries expressing germline *UASp-Act42D.GFP* with CytoD significantly reduced the number of niche-directed APs while nocodazole had no effect (Figure 1I). Conversely, treatment of *GFP.αTub*-expressing ovaries with both drugs resulted in a significant reduction in the ability of GSCs to generate cytocensors, in comparison with DMSO-treated ovaries. This suggests that APs form independently of the MT network; however, the formation of cytocensors is dependent on actin. In addition, APs are more abundant than cytocensors (Figure 1I). The relative abundance of APs and the requirement for actin polymerization for cytocensor formation suggests that APs may represent the primary structure from which cytocensors emanate.

### Projections Dynamically Probe the Niche Microenvironment

**Figure 2:**
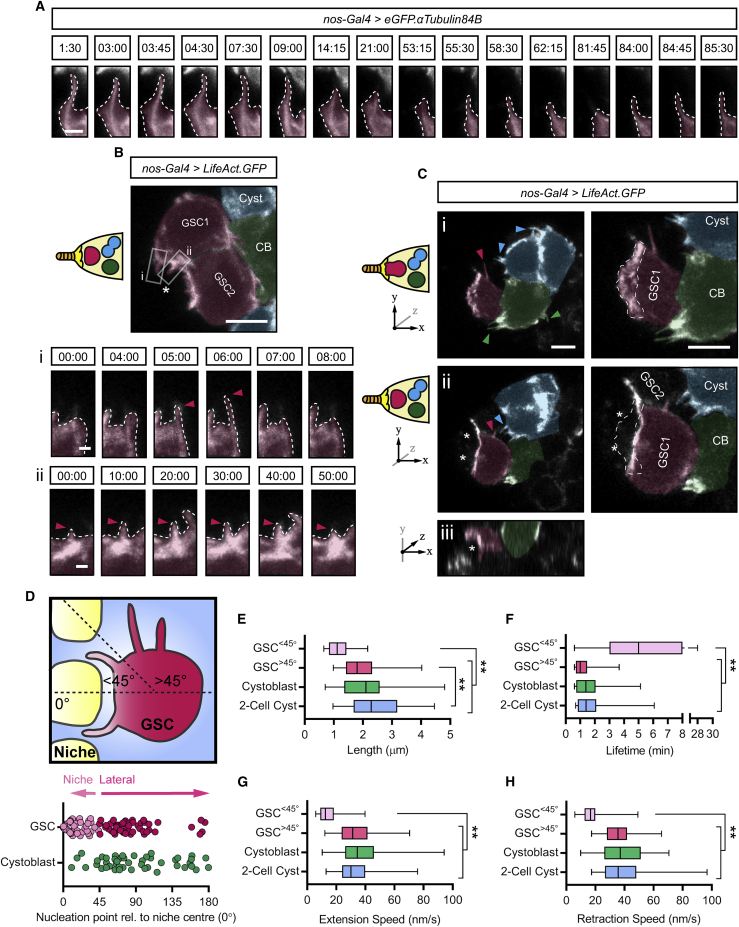
GSC Projections Are Dynamic, and All Early Germ Cells Form Actin-Rich Projections (A) Stills from Video S1 showing a cytocensor labeled with *eGFP.αTub* (false colored magenta) (time in min). Image only shows part of the GSC to enable visualization of the projection. (B) Stills from Video S2 showing F-actin with *LifeAct.GFP* in GSCs (false colored magenta), CBs (green) and 2- or 4-cell cysts (blue). (Bi) and (Bii) show stills of the regions in indicated boxes (time in min). Arrowheads indicate tip of the filopodium. (C) Same as (B) showing stills from Video S3. (Ci, left) The first two *z-*slices of a maximum projection showing the formation of F-actin-rich projections by GSCs, CBs, and cysts, indicated by boxes and color-coded arrowheads. (Ci, right) A closer view of the GSC in (Ci, left) showing a broad lamellipodia-like projection (outlined by dashed line) depicted in cartoon form on the left and axes denote position within the maximum projection. (Cii, left) Two *z-*slices in the middle of the maximum projection reveal two CpCs (^∗^) positioned below the lamellipodium. (Cii, right) A closer view of the GSC in (Cii, left) with the position of the overlying lamellipodium indicated by the dashed line. (Ciii) *xz*-plane view. (D) Cartoon and scatter plot showing the angle of actin filopodia nucleation point relative to the center of the niche (0°). “Niche-directed” filopodia are defined as those forming at an angle <45° to the center of the niche. The rest are defined as “lateral projections” (n = 100 GSC projections and n = 50 CB projections). (E–H) Box and whisker plots comparing the length (E), lifetime (F), extension (G), and retraction speed (H) of actin projections from the following classes: GSC^<45°^ (light pink), GSC^>45°^ (magenta), CBs (green), and 2-cell cysts (blue). Median, 25^th^ and 75^th^ percentile, and whiskers show minima and maxima. n = 46–50 projections from n ≥ 8 cells each. Dashed lines outline GSC projections. Scale bar, 2 μm (A) or 5 μm (B and C). ^∗∗^p < 0.0001. See also Figure S3.

The above data showing enrichment of tyrosinated MTs and the non-uniformity of α-tubulin acetylation suggest that the cytocensors may be dynamic in nature. To address this, we used live imaging to monitor MT dynamics in *GFP.αTub*-expressing GSCs *ex vivo*. GSCs extend cytocensors that dynamically probe the niche microenvironment over the course of 1–2 h (Video S1). Figure 2A shows stills from Video S1, which focus on a single motile cytocensor extended into the niche that collapses and reforms multiple times over the course of imaging. This shows that cytocensors are relatively dynamic in nature. We similarly visualized F-actin by driving germline expression of *UASp-LifeAct.eGFP* (Video S2). Strong labeling of cortical F-actin is seen at the GSC-niche interface from which the APs emerge (Figure 2B). The GSCs generate short filopodia-like projections that extend into the niche, and although they appear dynamic, a subset exhibits much longer lifetimes (compare the relatively short-lived AP in Figure 2Bi with a longer-lived one in Figure 2Bii). We also found additional transient lateral GSC projections (Figure 2C, magenta arrowhead; Video S3), while all early differentiating germ cells (CBs [green], 2- to 4-cell cysts [blue]) generate long, transient APs (Figures 2Ci and 2Cii). In addition, some GSCs extend broad lamellipodia-like projections that extend over or in between multiple niche cells (Figures 2Ci–2Ciii; Video S3), and individual finger-like filopodia extend from the ends of these structures to envelope niche CpCs (Figure S3A; Video S4). During mitosis, GSCs undergo typical cell rounding, accompanied by cortical F-actin accumulation and the collapse of APs, which rapidly reform as the GSCs re-establish contact with niche CpCs (Figure S3B; Video S5).

Video S1. A Dynamic Cytocensor Projecting into the Niche; Germline Expression of UASp-eGFP.αTub, Related to Figure 2 Video represents a maximum projection over 6 μm. Time = min:s at 5 frames per second. Scale bar indicates 5 μm.

Video S2. Dynamic-Actin-Based Filopodia Probe the Niche; Germline Expression of UASp-LifeAct.GFP, Related to Figure 2 Video represents a maximum projection over 6.75 μm. Time = min:s at 5 frames per second. Scale bar indicates 5 μm.

Video S3 All Early Germ Cells Form Dynamic Actin Projections; Germline Expression UASp-LifeAct.GFP, Related to Figures 2 and S2 Video represents a maximum projection over 3 μm. Time = min:s at 5 frames per second. Scale bar indicates 5 μm.

Video S4. Looking down the Shaft of a Filopodium Extending into the Niche from a Lamellipodium; Germline Expression of UASp-LifeAct.GFP, Related to Figures 2 and S2 Video represents a maximum projection over 1.5μm. Time = min:s at 5 frames per second. Scale bar indicates 5 μm.

Video S5. GSC Projections Collapse Prior to Mitosis and Rapidly Reform after Cell Division; Germline Expression of UASp-LifeAct.GFP, Related to Figures 2 and S2 Video represents a maximum projection over 1.5μm. Time = min:s at 5 frames per second. Scale bar indictes 5 μm.

GSCs appear to extend shorter projections toward the niche with lateral projections tending to be longer and more transient (Figure 2C; Video S3). To compare the nature of these projections, we grouped together those that extend into the niche (GSC^<45°^; point of nucleation occurs at <45° relative to the center of the niche [0°]) and those that extend laterally (GSC^>45°^; point of nucleation that occurs at >45° is defined as a lateral projection) and compared these to the projections generated by the differentiating cells (Figure 2D). GSC projection formation appears polarized, as most APs are nucleated in the direction of the niche, or below 90°, while CBs tend to generate projections at any angle >45° (Figure 2D). This may simply be due to structural hindrance, with the presence of neighboring GSCs precluding the formation of niche-directed CB projections. Comparing the length, lifetime, and speed of filopodia extension and retraction reveals that GSC^<45°^ projections are significantly shorter, slower, and more stable than GSC^>45°^ projections (Figures 2E–2H). These lateral projections appear similar to those formed by CBs and 2-cell cysts; they are generally longer, the extent of which increases as differentiation progresses (Figure 2E), and significantly more transient and/or unstable, indicated by their short lifetime (Figure 2F) and their speed of growth and collapse (Figures 2G and 2H). Together, these data show that GSC projections are dynamic and probe the niche. In addition, the formation of unstable APs is a common trait of early germ cells, while GSCs also extend more stable, short APs into the niche.

### Cytocensors Form in Response to Dpp Signaling

**Figure 3:**
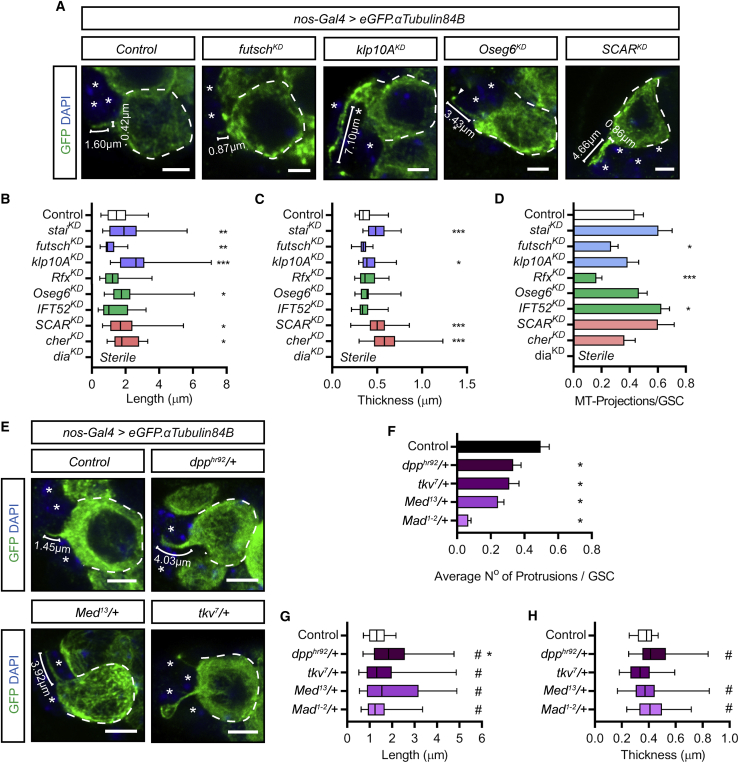
Cytocensors Are Regulated by Stem-Cell-Enriched MT-Associated Factors and Form in Response to Dpp Signaling (A) Germline-specific *shRNA* expression for the indicated MT-associated factors and actin regulators disrupts projection formation. (B and C) Box and whisker plots of cytocensor length (B) and thickness (C) following knockdown of the expression of MT-associated factors (blue), ciliogenesis factors (green), and actin regulators (red). Median, 25^th^ and 75^th^ percentile, and whiskers show minima and maxima. n ≥ 30 projections. (D) Bar chart showing number of cytocensors formed per GSC for knockdown of factors as in (B) and (C). Mean and SEM, n = 100 GSCs. (E–H) Heterozygous mutants for Dpp signaling pathway components, *dpp*^*hr92*^, *tkv*^*7*^, *Med*^*13*^, and *Mad*^*1-2*^, typically form abnormal cytocensors compared to controls, examples shown in (E). Reduced Dpp signaling disrupts projection formation (F), length (G), and/or thickness (H). Statistics as in (B)–(D). #, p < 0.01 F-test. Dashed lines outline individual GSCs. (^∗^) CpCs; brackets, length or thickness. Scale bars, 2 μm. ^∗^p < 0.05; ^∗∗^p < 0.001; ^∗∗∗^p < 0.0001. See also Figure S4.

Our RNA-seq data suggest that Dpp signaling activates *futsch* and *Rfx* expression, while GSCs also showed general enrichment of other cytoskeletal- and ciliogenesis-associated factors. We therefore determined whether a subset of these factors plays a role in the formation of GSC cytocensors. To achieve this, germline-specific RNAi was used to firstly address the roles of the MT-stabilizer Futsch and the tubulin-binding protein Stathmin (Stai). *futsch*^*KD*^ expression significantly reduces the frequency of cytocensors formed, while those that are formed are shorter than wild-type projections (Figures 3A–3D). Conversely, *stai*^*KD*^ expression leads to significantly longer and thicker projections. We also knocked down expression of Klp10A, a MT-depolymerizing kinesin shown to regulate MT-nanotube formation and centrosome size in male GSCs (Chen et al., 2016, Inaba et al., 2015). This also leads to significantly longer and slightly thicker projections than wild type (Figures 3A–3D). Klp10A does not, however, appear to regulate female GSC centrosome size (Figures S4A and S4Ai).

We next addressed the roles of three genes associated with ciliogenesis. *Rfx*^*KD*^ expression leads to a significant decrease in the frequency of cytocensor formation (Figure 3D), while those that are formed appear normal in length and thickness (Figures 3B and 3C). Two intraflagellar transport proteins were also included in our analysis, Oseg6 and IFT52, which are expressed in GSCs and CBs (Table S1) and function downstream of Rfx (Laurençon et al., 2007). *Oseg6*^*KD*^ expression results in longer cytocensors that can often be found to contain a globular accumulation of tubulin at the tip (Figure 3A), while *IFT52*^*KD*^ expression resulted in a small increase in the frequency of cytocensor formation. Together, these data identify a set of factors, two of which are up-regulated by Dpp signaling (Futsch and Rfx), that regulate cytocensor formation.

As actin is necessary for the formation of MT-rich cytocensors (Figure 1I), we examined three actin cytoskeletal components and regulators of filopodia and cytoneme formation: the formin Diaphanous (Dia), SCAR, and filamin (cheerio, cher). *dia*^*KD*^ expression results in complete loss of the germline; therefore, the effect on cytocensor formation could not be determined. However, knockdown of either *SCAR* or *cher* expression resulted in the formation of abnormally long and thick projections (Figures 3A–3C). These results further highlight the role of actin in the formation of cytocensors.

Two of the genes validated as regulators of cytocensor formation are *Rfx* and *futsch*, both of which were identified as positive Dpp targets in our RNA-seq data. This suggests that cytocensor formation is downstream of Dpp signaling in GSCs. To test the requirement for Dpp signaling, we visualized cytocensor formation in germaria from female flies heterozygous for mutations of the ligand, receptor (*tkv*), or Smads (*Mad* and *Med*). Heterozygotes were used as a sensitized background because analysis of homozygous mutants is not possible due to the rapid differentiation of GSCs in the absence of Dpp signaling. All heterozygotes show a significantly reduced ability to form projections, with *Mad*^*1-2*^/*+* causing the greatest loss (Figures 3E and 3F). In addition, cytocensors that are formed are frequently abnormal, particularly longer, and thicker than controls (Figures 3G and 3H). These results show that the frequency of cytocensor formation correlates with the ability of GSCs to receive and transduce Dpp signaling.

To further test the role of Dpp signaling in cytocensor formation, we exploited the previous observation that knockdown of ColIV expression in larval hemocytes, which deposit ColIV within the niche, results in increased Dpp signaling range and the accumulation of ectopic GSCs outside the niche (Van De Bor et al., 2015). Hemocytes deposit niche ColIV while contributing only little ColIV to the rest of the ovary. We therefore utilized this specificity to address whether ectopic Dpp could induce cytocensor formation. Wild-type germ cells that exit the niche, and therefore do not receive Dpp, do not typically generate cytosensors (Figure S4B). Prevention of ColIV deposition in the niche by larval hemocytes (*HmlΔ-Gal4* > *ColIV*^*KD*^) extends Dpp signaling range, resulting in the accumulation of ectopic GSC-like cells (Van De Bor et al., 2015) that extend cytocensors (Figure S4C). These data are consistent with Dpp signaling acting as a regulatory input for cytocensor formation.

### CpC Presentation of Dally Generates a Reservoir of Dpp

**Figure 4:**
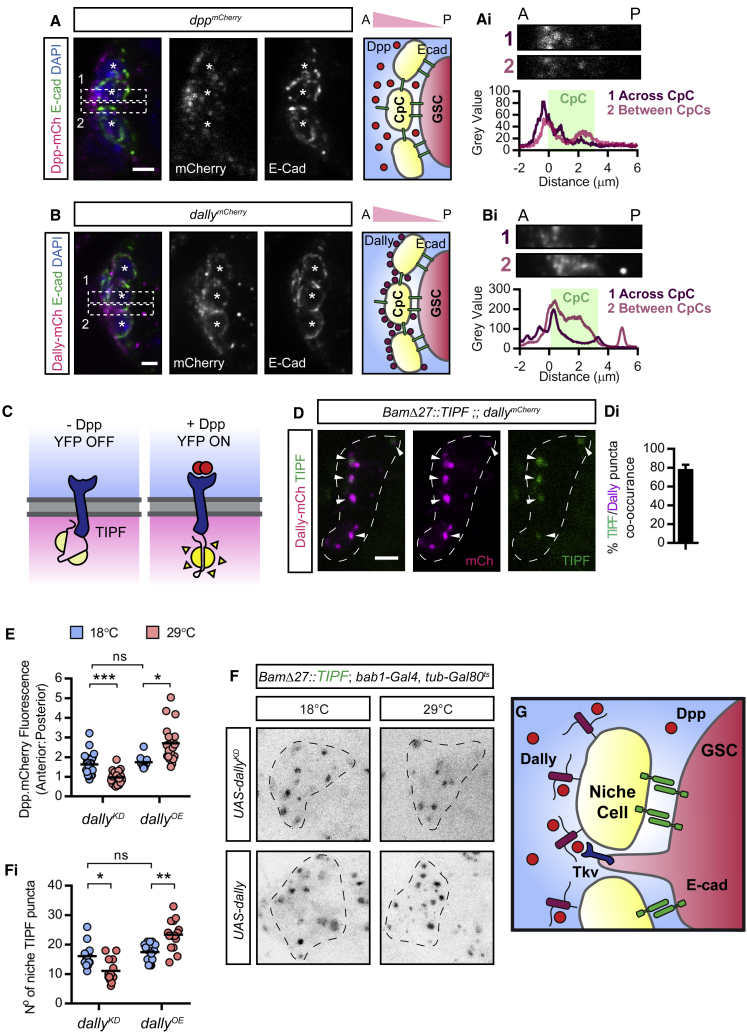
Niche Cells Create a Dally-Dpp Reservoir where GSC Tkv Activation Occurs (A and B) Extracellular staining of Ecad and endogenous mCherry-tagged Dpp (A) or Dally (B). Boxes show where the graphs of fluorescence intensity (Ai and Bi) were taken from anterior to posterior (A to P) through the center of the niche or between two CpCs (^∗^) as shown in the higher magnification views above. Ecad defines the niche cell boundaries (green). (C) Cartoon illustrating the TIPF reporter that fluoresces only upon ligand-receptor binding. (D) Endogenous fluorescence of Dally^mCh^ and germline-expressed TIPF reporter (*BamΔ27::TIPF*) around the niche. (Di) Percentage co-occurrence of TIPF and Dally^mCh^ puncta. Mean and SD. n = 20 germaria. (E) Graph shows anterior-to-posterior ratio of fluorescence intensity of Dpp^mCh^. *dally* knockdown (*dally*^*KD*^) or overexpression (*dally^OE^*) is induced at 29°C and compared to non-induced controls raised at 18°C. Line shows mean. n = 20 CpCs. (F) Endogenous fluorescence of TIPF reporter with *dally* knockdown or overexpression in inverted black and white for clarity. (Fi) Graph showing the number of TIPF puncta per germaria in (F). Line shows mean, n = 20 germaria. (G) Cartoon model showing that GSCs present Tkv on projections to access a Dally-Dpp reservoir. Dashed lines (D and G) outline the niche. Scale bar, 2 μm. ^∗^p < 0.01; ^∗∗^p < 0.001; ^∗∗∗^p < 0.0001. See also Figure S5.

To investigate the function of GSC-niche-directed projections, we examined the localization of Dpp around the niche using endogenous Dpp tagged with mCherry (Dpp^mCh^; Fereres et al., 2018), in the same position as the previously described Dpp^GFP^ (Entchev et al., 2000). These Dpp^mCh^ flies are homozygous viable and show no overt germarium phenotype (Figures S5A and S5B). Using extracellular staining of Dpp^mCh^ and Ecad, which outlines CpCs, we detect Dpp^mCh^ concentrated in puncta to the anterior of the niche creating an anterior-to-posterior high-to-low gradient (Figures 4A and 4Ai). This same localization pattern is also observed using two previously described Dpp transgenic lines tagged with either hemagglutinin (HA) (Figure S5C; Shimmi et al, 2005) or GFP (Figure S5D; Teleman and Cohen, 2000), which tag both forms of Dpp generated by pre-protein cleavage. This shows that secreted Dpp is concentrated away from GSCs, creating a short anteroposterior gradient across the niche.

The HSPG Dally is expressed by CpCs and promotes Dpp signaling in GSCs (Guo and Wang, 2009, Hayashi et al., 2009). Furthermore, Dally can bind Dpp and is proposed to regulate its extracellular distribution (Akiyama et al., 2008). We therefore visualized the localization of endogenous Dally tagged with mCherry (Dally^mCh^). Like Dpp, we find an anteroposterior gradient of punctate extracellular Dally^mCh^ (Figures 4B and 4Bi), suggesting that Dally binds Dpp and contributes to the formation of the Dpp gradient. However, this ligand distribution is incompatible with the classical view of Dpp signaling in the germarium, which predicts Dpp accumulation at the interface of GSCs and niche CpCs (Figure S1B). We therefore addressed where Tkv activation occurs using a fluorescent reporter of ligand-receptor interaction, TIPF (Figure 4C; Michel et al., 2011). TIPF is Tkv C-terminally tagged with YFP held in a non-fluorescent conformation, but upon ligand-receptor binding, the YFP is released and adopts a fluorescent conformation. Using the endogenous fluorescence of germline-expressed TIPF (*Bam27::TIPF*), we find the majority of active Tkv co-occurring with Dally^mCh^ (Figures 4D and 4Di). This suggests that Dally-bound Dpp is a key source of ligand for GSCs and that signaling likely occurs on GSC projections that are extended into the niche.

To address whether Dally regulates Dpp distribution around the niche, the Gal80^ts^ system was used to temporally induce *dally* knockdown (*dally^KD^*) or overexpression (*dally^OE^*) in niche cells. In adults raised at 18°C, Gal80^ts^ represses Gal4 activity and therefore expression of the associated transgene. Shifting adults to 29°C for 3 days causes Gal80^ts^ inactivation to enable transgene expression. When *dally*^*KD*^ or *dally*^*OE*^ adult flies are raised at 18°C, extracellular Dpp^mCh^ shows an anterior high gradient as seen in wild-type germaria (Figures S5E and S5F). Inducing *dally* knockdown results in more equal levels of Dpp across the niche (Figures 4E, S5E, and S5Ei). Conversely, *dally* overexpression leads to greater anterior accumulation of Dpp^mCh^, resulting in a steeper gradient with a higher average anterior-to-posterior ratio (Figures 4E, S5F, and S5Fi). Together, these results suggest that niche-expressed Dally binds and sequesters Dpp away from the GSCs forming a reservoir of Dpp.

To provide further evidence that Dally-bound Dpp is the source of ligand for GSCs, we used the TIPF reporter to monitor Tkv activation following manipulation of *dally* expression in niche cells, as described above. Induction of *dally* knockdown at 29°C decreases the number of TIPF puncta compared to controls (18°C; Figures 4F and 4Fi). Conversely, *dally* overexpression increases the number of niche TIPF puncta. These data are consistent with Dally-bound Dpp representing the major source of self-renewal signal for GSCs, while the extension of projections likely enables access (Figure 4G).

**Figure 5:**
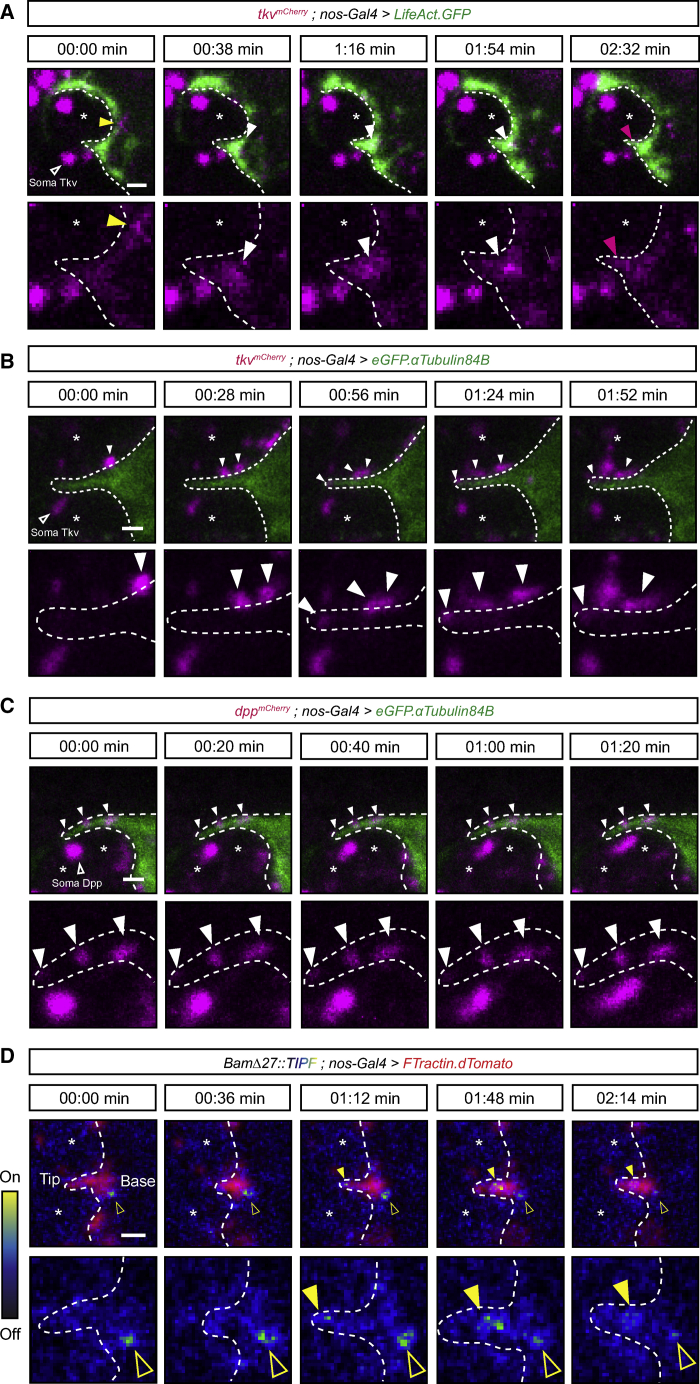
Tkv Is Trafficked onto GSC Projections that Act as Signaling Platforms (A) Stills from a video of F-actin labeled with LifeAct.GFP and endogenous mCherry-tagged Tkv showing Tkv^mCh^ (yellow arrowhead) at the GSC-niche interface before accumulating at the base (white arrowhead) and then upon the AP (magenta arrowhead). (Bottom) Shows close-up view of Tkv^mCh^ channel. (B) As in (A) showing the trafficking of Tkv^mCh^ puncta (white arrowheads) on a cytocensor labeled with eGFP.αTub*.* (Bottom) Shows close-up view of Tkv^mCh^ channel. (C) As in (B) showing Dpp^mCh^ puncta (white arrowheads) statically associated with a cytocensor labeled with eGFP.αTub. (Bottom) Shows close-up view of Dpp^mCh^ channel. (D) As in (A) showing active Tkv at the base of an FTractin-dTomato-labeled AP (TIPF fluorescence, open yellow arrowhead) and Tkv activation occurring on an AP (yellow arrowhead). (Bottom) Shows close-up view of TIPF. TIPF is false colored as a heatmap for clarity. Projections are outlined by dashed lines. (^∗^) CpCs. Scale bars, 1 μm. See also Figure S5.

To directly determine if GSC projections allow access to the anterior reservoir of Dpp, we used live *ex vivo* imaging to monitor Dpp and Tkv localization on GSC projections. An N-terminally tagged Tkv^YFP^ knock-in line (Lowe et al., 2014), while homozygous viable, was found to generate tumors of GSC-like cells (Figures S5A and S5B), suggesting that Tkv regulation is impaired. Conversely, a C-terminally tagged Tkv^mCh^ knock-in line is homozygous viable and displays no germline phenotype (Figures S5A and S5B) and so was used hereafter. Firstly, the localization of Tkv was monitored on APs using germline expression of *LifeAct.GFP* (Figure 5A). Tkv^mCh^ is detected at the GSC-niche interface (Figure 5A, yellow arrowhead) and around short APs (white arrowhead). Upon extension into the niche, Tkv^mCh^ localizes along the projection (magenta arrowhead), suggesting that APs could function as Dpp signaling platforms. In addition, Tkv^mCh^ also decorates lateral GSC projections (Figure S5G). Tkv^mCh^ was also visualized in *GFP.αTub*-expressing GSCs (Figure 5B). Here, large puncta of Tkv^mCh^ were detected trafficking onto cytocensors and accumulating at the tip. The trafficking of larger Tkv^mCh^ puncta could be indicative of the active trafficking of Tkv^mCh^ onto cytocensors (e.g., by vesicular transport) in comparison with APs, which may be more random. To determine whether these projections contact the Dpp reservoir, Dpp^mCh^ was visualized with eGFP.αTub (Figure 5C). Cytocensors form stable contacts with Dpp^mCh^ puncta. Finally, in order to determine whether Tkv activation occurs on GSC projections, we used germline expression of *UASp-FTractin.dTomato* to visualize actin filopodia alongside TIPF. In Figure 5D, a single TIPF puncta is localized to the base of a pre-existing filopodium. The projection collapses and at 1:12 min has reformed. At this point, a TIPF puncta appears at the tip of the AP and moves toward the base. GSC projections can therefore act as sites of Dpp signal transduction. Together, these data are consistent with the GSCs dynamically localizing Tkv onto cellular projections to permit access to Dpp.

### Dia Regulates Actin-Projection Formation and Dpp Signal Reception

**Figure 6:**
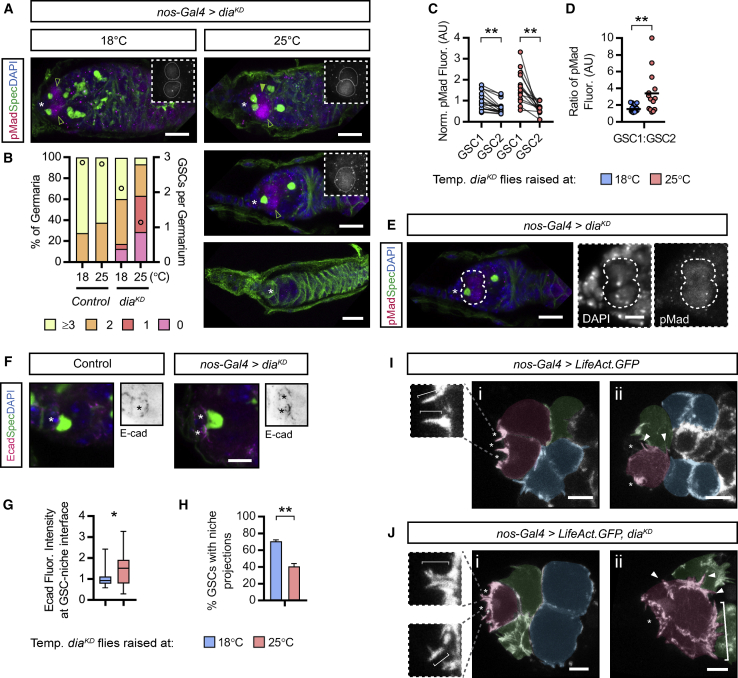
Knockdown of *dia* Expression Causes Dysregulation of Dpp Signaling and Actin Projection Formation (A) Germline-specific *dia*^*KD*^ expression. Comparison of adults raised at 18°C for 3 days (inhibiting *shRNA* expression) or at 25°C for 3 days (inducing *shRNA* expression). (Insets) pMad staining reports the Dpp signaling response. Early germ cells are marked by the presence of the spectrosome (αSpectrin). (B) Histogram showing quantification of GSC numbers in (A) n = 50 germaria for control, n ≥ 142 for *dia*^*KD*^. (C) Comparison of pMad fluorescence shown in (A). GSC1 is the cell with the higher pMad and GSC2 has the lowest. n > 15 germaria. (D) Ratio of pMad levels from (C). Line shows mean. (E) A single polyploid *dia*^*KD*^-expressing GSC that occupies an entire niche. (F and G) As in (A) showing (F) Ecad levels around the niche, also in inverted black and white for clarity, and (G) quantification in control (n = 27) and *dia*^*KD*^ (n = 32). Median, 25^th^ and 75^th^ percentile, and whiskers show minima and maxima. (H) Percentage of *dia*^*KD*^-expressing GSCs that form actin-rich projections labeled with LifeAct.GFP. Mean and SD of n > 100 GSCs; 3 biological replicates. (I and J) Stills showing actin projections labeled with LifeAct.GFP in (I) wild-type and (J) *dia*^*KD*^-expressing germ cells. Brackets in (Jii) label supernumerary projections and arrowheads label lateral projections. GSC (false colored magenta), CB (green), and 2- or 4-cell cysts (blue). (^∗^) CpCs. Scale bars, 5 μm (A and F) or 2 μm (G and H). ^∗^p < 0.05; ^∗∗^p < 0.001.

As Dpp signal transduction is observed on GSC projections, we next wanted to test their requirement for Dpp signaling. We first returned to the actin polymerizing factor Dia, which is necessary for GSC maintenance (Figure 3). By raising *dia*^*KD*^ flies at 18°C, *shRNA* expression is repressed during larval development (Figures 6A and 6B). Adults maintained at 18°C for 3 days do exhibit some GSC loss, suggesting that there is leaky *shRNA* expression and highlighting the sensitivity of GSCs to *dia* levels. When adults are shifted to 25°C for 3 days, rapid GSC loss is observed (Figures 6A and 6B), with many germaria containing single (Figure 6A, middle panel) or no GSCs (bottom panel). To determine if GSC loss is due to perturbed Dpp signaling, we visualized pMad. When *dia* expression is knocked down, we do not observe a loss of pMad; instead, neighboring GSCs begin exhibiting greater variability in pMad levels. Typically, one GSC per niche experiences slightly higher levels of pMad than its neighbor (Figure 6A, top panel; Figures 6C and 6D). This disparity may be due to uneven knockdown in neighboring GSCs. On the other hand, previous studies have shown that Dpp signaling mutant clones are outcompeted by neighboring wild-type GSCs and replaced by symmetric cell division (Xie and Spradling, 1998). The disparity in pMad levels seen with *dia*^*KD*^ could similarly be expected to promote such competition and lead to a “winner” higher pMad GSC outcompeting the other. However, if this occurs here, we speculate that the block on cytokinesis due to low *dia* leads to single large polyploid “winner” GSCs that occupy entire niches (Figure 6E). We note that other functions of Dia could also contribute to GSC loss, such as polyploidy. We rule out that GSC loss is due to reduced niche adhesion as Ecad levels at the GSC-niche interface increase following *dia* knockdown (Figures 6F and 6G) consistent with disrupted endocytosis (Levayer et al., 2011; see below). Regardless of mechanism, we show that reduced *dia* expression leads to enhanced Dpp signaling in a subset of GSCs.

We next determined whether Dia regulates AP formation using *LifeAct.GFP* coexpression. Upon *dia* knockdown, the number of GSCs extending projections into the niche is decreased (Figure 6H), whereas the projections that remain are abnormal. While wild-type projections are thin, finger-like filopodia (Figure 6Ii), or broader lamellipodia (Figure 2C), *dia*^*KD*^ GSCs extend branched, thick projections (Figure 6Ji). Furthermore, their formation appears disorganized with the extension of supernumerary lateral projections (compare the APs [arrowheads] in Figure 6Iii and the APs [arrowheads] and lamellipodial projections [bracket] in Figure 6Jii). These results suggest that enhancing GSC projection formation could increase Dpp signal transduction.

### Cytoskeletal Projections Are Necessary for Dpp Signal Activation and Attenuation

**Figure 7:**
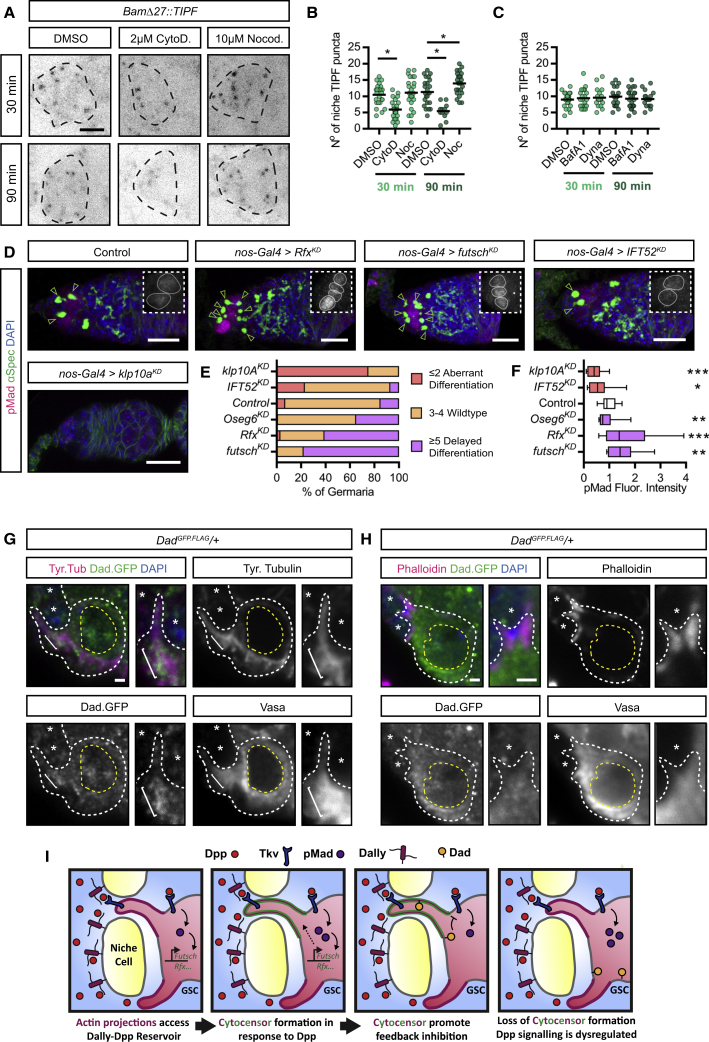
Cytocensors Attenuate Dpp Signaling to Regulate GSC Self-Renewal and Differentiation (A) Endogenous TIPF fluorescence after 30 or 90 min *ex vivo* drug treatment. (B and C) Scatter plots showing number of TIPF puncta per niche after treatment with (B) DMSO (n = 24 each), 2 μM CytoD (n = 28 and 12, respectively), and 10 μM nocodazole (n = 24 each) and (C) DMSO (n = 24 and 19, respectively), 100 nM BafA1 (n = 22 and 23, respectively), and 100 μM dynasore (n = 19 each). Line shows mean. (D) Germline-specific *shRNA e*xpression phenotypes. (Insets) pMad staining reports the Dpp signaling response. Early germ cells are marked by the presence of the spectrosome (open arrowhead) labeled by anti-αSpectrin. (E) Histogram showing quantification of GSC numbers in (B) and differentiation phenotype. n ≥ 100 germaria. (F) Comparison of pMad fluorescence relative to controls in (D). Median, 25^th^ and 75^th^ percentile, and whiskers show minima and maxima. n ≥ 15 cells. (G and H) Immunofluorescence staining of a *Dad*^*GFP.FLAG*^*/+* germarium stained for (G) tyrosinated α-tubulin to label cytocensors or (H) phalloidin to label APs. White dashed line shows individual GSCs and yellow dashed line outlines the nucleus. Bracket indicates Dad.GFP concentrated at the base of a cytocensor. (I) Model illustrating the role of APs in promoting Dpp signal transduction and cytocensors in modulating levels through signal suppression. See text for details. Dashed lines in (A) outline the niche. Scale bar, 10 μm (B), 5 μm (A), or 1 μm (G). ^∗^p < 0.05; ^∗∗^p < 0.001; ^∗∗∗^p < 0.0001. See also Figures S6 and S7.

If GSC projections are necessary for accessing the Dpp reservoir, inhibiting projection formation would be predicted to compromise Dpp signal reception, transduction, and GSC fate. We first tested whether inhibiting projection formation with chemical inhibitors (Figure 1I) disrupted receipt of Dpp in GSCs using the number of TIPF puncta around the niche as a readout. Germaria incubated *ex vivo* with DMSO maintained similar levels of signal activation after 30 and 90 min (Figures 7A and 7B). Incubation with CytoD, however, results in a significant reduction in TIPF puncta even after 30 min (Figures 7A and 7B) in agreement with a role for projections in Dpp signal reception. Incubation with nocodazole for 30 min has no effect on the average number of TIPF puncta (Figures 7A and 7B), suggesting that in the absence of cytocensors, GSCs are still able to access and receive Dpp through APs (Figure 1I). However, there is a small but significant increase in the number of TIPF puncta following a 90-min nocodazole treatment (Figures 7A and 7B). The overactive signaling observed in the absence of cytocensors raises the possibility that in contrast to APs, they are necessary for signal attenuation.

An alternative interpretation of these data is that interfering with actin/MT polymerization disrupts receptor endocytosis, trafficking, and/or degradation, all of which can influence BMP/Dpp signaling output (Ehrlich, 2016). To test this, we assessed the disruption to trafficking in the drug treatment time frame and whether this would be sufficient to give the observed changes in TIPF puncta numbers (Figures 7A and 7B). First, we carried out a dextran uptake assay using fluorescently tagged dextran as a fluid-phase marker (Figures S6A–S6E). While dextran uptake is readily observed in controls (Figure S6A), treatment with CytoD greatly reduces uptake (Figure S6B), consistent with observations that inhibiting actin polymerization blocks endocytosis (Mortensen and Larsson, 2003). A similar result is obtained following treatment with Dynasore (Figure S6C), a potent inhibitor of dynamin-dependent endocytosis (Macia et al., 2006). Following nocodazole treatment, we observe increased dextran accumulation in smaller vesicles (Figure S6D), consistent with the inhibition of MT polymerization disrupting endosomal trafficking and/or maturation (Bayer et al., 1998). A similar result is observed upon treatment with bafilomycin A1 (BafA1) (Figure S6E), an inhibitor of endosomal-lysosomal maturation.

As these data show that incubating ovaries with CytoD and nocodazole perturbs endocytosis and trafficking, we next determined whether these additional effects of disrupted actin/MT polymerization could account for the altered Tkv activation (Figures 7A and 7B). To this end, we assayed the affect of Dynasore and BafA1 treatment on the number of niche TIPF puncta. In both cases, we observe no effect after 30 or 90 min of treatment (Figures 7C and S6F). These data suggest that the reduced or enhanced Tkv activation observed in Figures 7A and 7B is not due to reduced Tkv endocytosis, trafficking or degradation.

To complement the above data, we specifically investigated the effect of CytoD and nocodazole treatment on the endocytosis of Tkv^mCh^ by monitoring its co-localization with Rab5^YFP^, as early endosomal trafficking of Tkv in Rab5^+^ endosomes is known to enhance Dpp signal response (Gui et al., 2016). Only a small proportion of the Rab5^+^ endosomes per GSC are also Tkv^mCh^ positive in controls (Figures S6G–S6I). Incubation with CytoD significantly decreases the number of Rab5^+^ endosomes, indicative of inhibited endocytosis (Figures S6G and S6H; Mortensen and Larsson, 2003). However, this has little effect on the average number of Tkv^mCh^-positive Rab5^YFP^ puncta per GSC (Figure S6I) compared to the control. Incubation with nocodazole significantly increased the number of Rab5^+^ endosomes (Figure S6H); however, this also had little effect on the average number of Tkv^mCh^-positive Rab5^YFP^ puncta per GSC (Figure S6I). These data provide further evidence that the effect of CytoD or nocodazole on early endosomal trafficking of Tkv is insufficient to explain the altered Tkv activation observed.

We also addressed whether inhibition of actin polymerization leads to the loss of Ecad-based AJs, which are necessary for GSC maintenance (Song et al., 2002). Following 90 min of CytoD or nocodazole treatment, GSCs maintain contact with the niche CpCs (Figure S6J). Additionally, the spectrosome remains anteriorly anchored to the niche interface suggesting that polarity is maintained (Figure S6J; Deng and Lin, 1997). Nevertheless, we tested the effect of disrupting Ca^2^^+^-dependent cadherin binding by incubating germaria *ex vivo* with the Ca^2+^ chelator EGTA. A 90-min treatment with EGTA induces germline Ecad internalization (Figure S6K). However, EGTA treatment has no effect on the number of TIPF puncta per niche after 90 and even 150 min (Figures S6L and S6M). This shows that reduced niche adhesion is not sufficient to reduce Dpp signal transduction. We therefore favor the conclusion that reduced TIPF activation following CytoD treatment is due to loss of projections rather than its effect on Tkv trafficking or GSC adhesion.

Next, we further investigated cytocensor function through genetic manipulation by disrupting their formation through the germline-specific knockdown of factors described in Figure 3. pMad staining and the number of early germ cells were used to assay effects on Dpp signal transduction and GSC maintenance, respectively. Knockdown of *IFT52* or *klp10A*, which increases the frequency of projection formation or enhances the growth of cytocensors, respectively, leads to an increased rate of GSC loss (Figures 7D and 7E) and decrease in pMad levels (Figure 7F). This suggests GSCs aberrantly differentiate due to reduced Dpp signaling. We also observe a decrease in Ecad levels at the GSC-niche interface in *klp10*^*KD*^ GSCs (Figures S7A and S7B). However, it is not possible to determine whether this is a differentiation-associated loss (Shen et al., 2009) or due to a cytocensor-independent function of Klp10A in Ecad localization, although we favor the former as we observed continued Dpp signaling following loss of AJs by EGTA treatment (Figures S6L and S6M). We speculate that enhancing cytocensor formation or length is detrimental to the maintenance of pMad activation, such as through mis-trafficking of Tkv on long projections. However, as kinesin-like proteins have also been shown to shuttle endosomes, we cannot exclude that defective endosomal trafficking contributes to the lower pMad level in GSCs and their loss.

A weak delay in differentiation, typified by accumulation of ectopic early germ cells, is observed upon knockdown of *Oseg6* (Figure 7E). A stronger phenotype is observed upon knockdown of *futsch* or *Rfx* (Figures 7D and 7E), which both disrupt cytocensor formation (Figure 3). Consistent with this delayed differentiation, in both cases we detect a significant increase in GSC pMad levels (Figure 7F). These data show that when cytocensor formation is disrupted by knockdown of specific genes or chemical inhibition, Dpp reception is enhanced suggesting that cytocensors have an additional function, which is to attenuate Dpp signaling.

An alternative interpretation of the above data is that genetic knockdown of the regulators leads to a prolonged disruption of endocytosis, which alters GSC-CB fates through impaired BMP signal activation in GSCs and/or altered Ecad accumulation at the GSC-niche interface. We first addressed this by measuring dextran uptake following knockdown of cytocensor-regulatory factors. Knockdown of *dia*, a known regulator of endocytosis (Levayer et al., 2011), reduces dextran uptake (Figures S7C and S7D). However, no reduction or gross effect on dextran uptake is observed upon knockdown of the cytocensor regulators. Nonetheless, we directly addressed the effect of genetically inhibiting endocytosis on GSC-CB fates. We used germline knockdown of *shibire* (*shi*), encoding dynamin, to inhibit dynamin-dependent endocytosis (Figures S7E and S7F). This results in delayed differentiation of cysts and accumulation of cysts and developing nurse cells (yellow dashed line) within germaria (Figure S7E). *shi*^*KD*^ efficiently inhibits dextran uptake in GSCs (Figures S7C and S7D) and results in increased Ecad levels at the GSC-niche interface (Figures S7G and S7H), consistent with reduced Ecad endocytosis, but no change in pMad levels (Figures S7E and S7F). Together, these data suggest that the Dpp signal response in GSCs is independent of receptor endocytosis, while conversely, GSC-niche adhesion and germ cell differentiation are regulated by dynamin. As increased Ecad does not alter GSC number (Pan et al., 2007) and the phenotypes associated with knockdown of cytocensor regulators in Figure 7 are associated with alterations in pMad levels, we favor the interpretation that the phenotypes are due to impaired reception or attenuation of the Dpp signal rather than secondary effects on endocytosis.

We also investigated whether Rfx and Futsch could regulate the BMP signaling response between GSCs and their daughters independent of cytocensors. GSCs undergo an unusual cell cycle with delayed cytokinesis such that the GSC remains attached to its daughter pre-cystoblast (pCB) until G2 phase. Tkv activity is terminated in the pCB through the asymmetric upregulation of the kinase Fused, which is essential for differentiation by targeting Tkv for degradation (Xia et al., 2010, 2012). This results in the generation of a pMad gradient between the GSC-pCB with 20%–30% of pCBs displaying half the level of pMad observed in the GSC, whereas the majority of pCBs rapidly reduce pMad levels to less than a quarter (pMad^−^) of that of the GSC to enable differentiation (Figures S7I–S7K; Xia et al., 2012). Upon knockdown of *futsch* or *Rfx*, we observe more pMad^+^ pCBs (Figures S7I–S7K); however, the pMad gradient (relative pMad ratio between GSC and pCB) remains the same as observed for pMad^+^ pCBs in control germaria. This suggests that following *futsch* or *Rfx* knockdown, the circuitry regulating Dpp signal termination in pCBs remains functional, while the GSC exhibits enhanced Dpp signal reception. We therefore propose that the regulation of the Dpp signal response in GSCs by Futsch and Rfx is more likely through the promotion of cytocensor formation, which attenuates signaling levels.

Finally, to address how Dpp signaling may be attenuated we tested the hypothesis that the cytocensor provides a compartment for the coordinated concentration of both signaling machinery and antagonists. The classic Dpp target gene and inhibitory Smad, Dad, is expressed in GSCs and reduces Dpp signaling levels (Casanueva and Ferguson, 2004, Xie and Spradling, 1998). Immunofluorescence staining of transgenic GFP-tagged Dad shows that it is diffuse throughout the cytoplasm and nucleus and concentrates at the base of cytocensors (Figure 7G) but not APs (Figure 7H), consistent with a cytocensor-specific signal-attenuating property. In conclusion, we propose that the role of GSC projections is 2-fold: projections allow the receipt of secreted Dpp held away from the GSCs within a niche reservoir, while cytocensors enable the concerted localization of signaling machinery and antagonist(s) to modulate signaling levels (Figure 7I).

## Discussion

Here, we present data describing the changing transcriptome of GSCs as they transition from self-renewal to differentiation. Genes up-regulated in GSCs are associated with gonad development, chromatin organization, and transcriptional regulation. This is consistent with germline differentiation being accompanied by a reduction in transcriptional activity and altered chromatin organization (Flora et al., 2018, Zhang et al., 2014). Genes up-regulated in CBs include those encoding factors involved in cellular metabolism, growth, and protein production. This can be rationalized with CB biology, as upon exiting the stem cell niche, the CB quickly undergoes 4 rounds of mitosis to generate a 16-cell cyst, and this transition is associated with an increase in general and mitochondrial protein synthesis (Sanchez et al., 2016, Teixeira et al., 2015). We also identified Dpp target genes in GSCs and have elucidated the function of two positive Dpp targets, *Rfx* and *futsch*, which promote synthesis of cytocensors along with other MT-associated genes in the GSC transcriptome.

Based on our data, we propose a model whereby GSCs synthesize APs that access a Dally-bound reservoir of Dpp (Figure 7I). In support of this, short-term inhibition of AP formation significantly reduces active Tkv levels, whereas increasing AP branching and disorganization (*dia*^*KD*^) is associated with increased pMad levels. We propose that following activation of Dpp target genes, such as *Rfx* and *futsch*, these APs develop into cytocensors (Figure 7I) and provide evidence that, like the APs, these cytocensors access Dpp localized away from the GSCs.

GSC projections act at a much shorter range than typically associated with signaling filopodia, which are associated with the regulation of long-range signal transduction in many contexts. For example, cytonemes in the larval wing disc and dorsal air sac primordium drive long-range Dpp signaling (Wilcockson et al., 2017). The advantage of the unusual GSC-niche architecture may be 2-fold. Firstly, it concentrates the potent self-renewal signal further away from the GSCs, guarding against ectopic Dpp diffusion that would disrupt GSC differentiation. Secondly, and we suggest more importantly, our data provide evidence that the cytocensors allow GSCs to actively regulate their Dpp signaling levels by both collecting Dpp and attenuating signal transduction. Genetic or chemical perturbation of cytocensors leads to increased active Tkv and pMad (Figure 7I), suggesting that these cytocensors promote feedback inhibition to maintain signal transduction at a threshold that facilitates differentiation following GSC division. Mathematical modelling has previously shown that the pMad concentration prior to division is important so that levels fall below a critical threshold in the GSC daughter, allowing *bam* derepression and differentiation (Harris et al., 2011).

We speculate that cytocensors facilitate Dpp signal termination by acting as a hub where GSCs can concentrate signaling machinery and antagonists, such as Dad, to efficiently modulate pMad levels. Similarly, the Dad homolog Smad7 has been shown to localize to the base of primary cilia where it is proposed to inhibit TGFβR-Smad interaction to modulate signaling levels (Pedersen et al., 2016). This signal-attenuating activity of female cytocensors is in stark contrast to the role of male MT nanotubes, which increase the stem-cell-niche interface area to promote Dpp signal transduction. Perturbing nanotube formation decreases pMad levels, increasing the rate of competition-induced loss. Furthermore, male and female projections exhibit several structural differences. Male projections are MT-based static structures formed independently of actin, and most GSCs (∼80%) extend one or more of these projections into the body of neighboring niche cells (Inaba et al., 2015). Female cytocensors, on the other hand, are rich in both MTs and actin, are relatively dynamic, transient structures, and are much less frequently found (∼40% of GSCs). However, we propose that all GSCs will synthesize cytocensors, but at different times as they form in response to increasing Dpp signaling levels as part of a feedback mechanism. The distinct nature of male and female projections may be due to differing requirements for Dpp signaling in stem cell maintenance or more limiting Dpp levels and the competition between GSCs and somatic cyst stem cells for niche occupancy in the testis (Greenspan et al., 2015).

Given our evidence for signal transduction through GSC projections, this raises the question as to whether GSCs can receive Dpp in their absence. Monitoring Dpp-Tkv interaction following CytoD treatment shows that TIPF puncta are still present up to 90 min after treatment. This could be due to incomplete loss of APs or may suggest that diffusing Dpp drives low-level signal transduction. Alternatively, the maintenance of a few puncta could be due to perdurance of the TIPF reporter or disrupted endocytosis or trafficking of Tkv that was active prior to treatment (Lamaze et al., 1997). The identification of additional actin regulators involved in this process will be necessary to determine the absolute requirement for APs in GSC Dpp signaling. One putative factor is the small GTPase Rac1, a key regulator of the formation and identity of APs, which concentrates at the GSC-niche interface (Lu et al., 2012). Rac1 is necessary for long-term GSC maintenance and was suggested to promote BMP signal transduction.

We also find lateral GSC and CB projections decorated with Tkv^mCh^. It has been shown that differentiating germ cells remain highly sensitive to “leaky” Dpp, while ectopic Dpp can induce germ cell dedifferentiation (Xie and Spradling, 1998). The formation of these projections may therefore promote the sensitization of germ cells to Dpp signaling, enabling their dedifferentiation to replace GSCs lost during aging or stress (Liu et al., 2015).

Dynamic signaling projections enable the receipt or delivery of signaling molecules over large distances or between cells of different tissues. The ability of signaling projections to modulate signal transduction may be of particular importance to adult stem cells. These are cells that need to be sensitive to local and systemic signaling while still maintaining their own plasticity. While signaling projections are common during development, it remains to be determined whether they are also frequently found in adult stem cells. However, in the murine gut, intestinal stem cells extend apical processes that reach in between neighboring Paneth cells that constitute a key part of the intestinal stem cell niche (Barker et al., 2007) and Lgr4/5 has been shown to drive formation of cytoneme-like projections *in vitro* (Snyder et al., 2015). It will be interesting to determine the role of these projections in stem cell signaling and whether cytocensors are found in other developmental contexts.

## STAR★Methods

### Key Resources Table

**Table:** 

Reagent or Resource	Source	Identifier
**Antibodies**		

Rabbit polyclonal anti-GFP	Abcam	Cat# ab6556; RRID: AB_305564
Goat polyclonal anti-GFP	Abcam	Cat# ab6673; RRID: AB_305643
Rabbit polyclonal anti-mCherry	Abcam	Cat# ab183628; RRID: AB_2650480
Mouse monoclonal anti-RFP	Abcam	Cat# ab65856; RRID: AB_1141717
Rat monoclonal anti-αtubulin [YL1/2]	Abcam	Cat# ab6160; RRID: AB_305328
Mouse monoclonal anti-acetylated αtubulin	Abcam	Cat# ab24610; RRID: AB_448182
Rabbit monoclonal anti-Smad3 (phospho S423 + S425) [EP823Y]	Abcam	Cat# ab52903; RRID: AB_882596
Mouse monoclonal anti-γtubulin, Clone GTU-88	Sigma	Cat# NB 100-1628; RRID: AB_523854
Rabbit polyclonal anti-Vasa (d-260)	Santa Cruz	Cat# sc-30210; RRID: AB_793874
Mouse monoclonal anti-αSpectrin	DSHB	Cat# 3A9 (323 or M10-2); RRID: AB_528473
Rat monoclonal anti-E-cadherin	DSHB	Cat# DCAD2; RRID: AB_528120
Mouse monoclonal anti-futsch	DSHB	Cat# 22c10; RRID: AB_528403
Mouse Anti-HA Monoclonal Clone 12CA5	Roche	Cat# 11666606001; RRID: AB_514506
Donkey anti-Rabbit IgG Secondary Antibody, Alexa Fluor 488	Thermofisher	Cat# R37118; RRID: AB_2556546
Donkey anti-Goat IgG Secondary Antibody, Alexa Fluor 488	Thermofisher	Cat# A-11055; RRID: AB_2534102
Donkey anti-Mouse IgG Secondary Antibody, Alexa Fluor 555	Thermofisher	Cat# A-31570; RRID: AB_2536180
Donkey anti-Rabbit IgG Secondary Antibody, Alexa Fluor 555	Thermofisher	Cat# A-31572; RRID: AB_162543
Donkey anti-Rat IgG Secondary Antibody, Alexa Fluor 594	Thermofisher	Cat# A-21209; RRID: AB_2535795
Donkey anti-Rabbit IgG Secondary Antibody, Alexa Fluor 647	Thermofisher	Cat# A-31573; RRID: AB_2536183
Chicken anti-Rat IgG Secondary Antibody, Alexa Fluor 647	Thermofisher	Cat# A-21472; RRID: AB_2535875

**Chemicals, Peptides, and Recombinant Proteins**		

Collagenase type IV	Worthington Biochemicals	LS004186
TRIzol	Invitrogen	15596-018
Texas Red-X Phalloidin	Invitrogen	T7471
Prolong Gold Antifade with DAPI	Invitrogen	P36935
Dextran, Texas Red, 3000 MW, Neutral	Invitrogen	D3329
Fetal bovine serum	Sigma	F3018
Penicillin-Streptomycin	Sigma	P0781
Human insulin	Sigma	I2643
Paclitaxel	Sigma	T7402
Nocodazole	Sigma	SML1665
Cytochalasin D	Sigma	C2618
Bafilomycin A1	Sigma	B1793
Dynasore	Sigma	D7693
Schneider's Drosophila Medium	Thermofisher	21720-024
Fibrinogen, Bovine Plasma	Millipore	341573
Thrombin protease	GE Healthcare Lifesciences	27-0846-01

**Experimental Models: Organisms/Strains**		

*D. melanogaster*; *w^∗^;* *P{w+mC=vas.EGFP.HA}2*	DDGR (Kyoto)	Cat# 109171; RRID:DGGR_109171
*D. melanogaster*; *w^∗^;* *bam-GFP*	Chen and McKearin, 2003	N/A
*D. melanogaster*; *w^∗^;* *UASp-tkv*^*QD*^*/TM6B*	Tanimoto et al., 2000	N/A
*D. melanogaster*; *w^∗^;* *dally.mCh*	Norman et al., 2016	N/A
*D. melanogaster*; *w^∗^*, *Bam*Δ*27::TIPF*	Michel et al., 2011	N/A
*D. melanogaster*; *dpp*^*HA*^*/TM2*	Shimmi et al., 2005	N/A
*D. melanogaster*; *dpp*^*mCh*^	Fereres et al., 2018	N/A
*D. melanogaster*; *tkv*^*mCh*^	gift from T. Kornberg	N/A
*D. melanogaster*; *w^∗^;* *GAL4::VP16-nos*	Bloomington	Cat# 4937; RRID: BDSC_4937
*D. melanogaster*; *dpp[hr92] cn[1] bw[1]/SM6a*	Bloomington	Cat# 2069; RRID: BDSC_2069
*D. melanogaster*; *tkv[7] cn[1] bw[1] sp[1]/CyO*	Bloomington	Cat# 106937; RRID: DGGR_106937
*D. melanogaster*; *w[^∗^]; Mad[1-2] P{ry[+t7.2]=neoFRT}40A/CyO*	Bloomington	Cat# 7323; RRID: BDSC_7323
*D. melanogaster*; *ru[1] h[1] P{ry[+t7.2]=neoFRT}82B sr[1] e[s] Med[13]/TM3, Sb[1]*	Bloomington	Cat# 7340; RRID: BDSC_7340
*D. melanogaster*; *w[1118]; PBac{y[+mDint2] w[+mC]=Dad-GFP.FLAG}VK00037*	Bloomington	Cat# 42669; RRID: BDSC_42669
*D. melanogaster*; *w[^∗^]; P{w[+mC]=UAS-dpp.GFP.T}3/TM3, Sb[1]*	Bloomington	Cat# 53716; RRID: BDSC_53716
*D. melanogaster*; *w[^∗^] P{w[+mC]=UAS-dally.J}SJ1*	Bloomington	Cat# 5397; RRID: BDSC_5397
*D. melanogaster*; *w[^∗^]; P{w[+mC]=UASp-GFP.Act42A}5-5*	Bloomington	Cat# 9252; RRID: BDSC_9252
*D. melanogaster*; *M{w[+mC]=UASp-LifeAct.mGFP6}ZH-2A, w[^∗^]*	Bloomington	Cat# 58717; RRID: BDSC_58717
*D. melanogaster*; *P{w[+mC]=UASp-Act5C.mRFP}13, w[^∗^]*	Bloomington	Cat# 24777; RRID: BDSC_24777
*D. melanogaster*; *w[1118]; P{w[+mC]=GAL4::VP16-nos.UTR}CG6325[MVD1], P{w[+mC]=UASp-GFPS65C-alphaTub84B}3*	Bloomington	Cat# 7253; RRID: BDSC_7253
*D. melanogaster*; *w[^∗^]; sna[Sco]/CyO; P{w[+mC]=UASp-F-Tractin.tdTomato}10C/TM2*	Bloomington	Cat# 58988; RRID: BDSC_58988
*D. melanogaster*; *UASp-bam*^*shRNA*^ *y1 v1; P{TRiP.HMS00029}attP2*	Bloomington	Cat# 33631; RRID: BDSC_33631
*D. melanogaster*; *UASp-futsch*^*shRNA*^ *y[1] v[1]; P{y[+t7.7] v[+t1.8]=TRiP.HMS02000}attP40*	Bloomington	Cat# 40834; RRID: BDSC_40834
*D. melanogaster*; *UASp-Rfx*^*shRNA*^ *y[1] v[1]; P{y[+t7.7] v[+t1.8]=TRiP.HMJ23335}attP40*	Bloomington	Cat# 61847; RRID: BDSC_61847
*D. melanogaster*; *UASp-klp10A*^*shRNA*^ *y[1] sc[^∗^] v[1]; P{y[+t7.7] v[+t1.8]=TRiP.HMS00920}attP2*	Bloomington	Cat# 33963; RRID: BDSC_33963
*D. melanogaster*; *UASp-dia*^*shRNA*^ *y[1] sc[^∗^] v[1]; P{y[+t7.7] v[+t1.8]=TRiP.GL00408}attP40/CyO*	Bloomington	Cat# 35479; RRID: BDSC_35479
*D. melanogaster*; *UASp-SCAR*^*shRNA*^ *y[1] sc[^∗^] v[1]; P{y[+t7.7] v[+t1.8]=TRiP.HMS01536}attP40*	Bloomington	Cat# 36121; RRID: BDSC_36121
*D. melanogaster*; *UASp-cher*^*shRNA*^ *y[1] sc[^∗^] v[1]; P{y[+t7.7] v[+t1.8]=TRiP.HMS01501}attP2/TM3, Sb[1]*	Bloomington	Cat# 35755; RRID: BDSC_35755
*D. melanogaster*; *UASp-nuf*^*shRNA*^ *y[1] sc[^∗^] v[1]; P{y[+t7.7] v[+t1.8]=TRiP.HMS02713}attP2*	Bloomington	Cat# 43999; RRID: BDSC_43999
*D. melanogaster*; *UASp-stai*^*shRNA*^ *y[1] sc[^∗^] v[1]; P{y[+t7.7] v[+t1.8]=TRiP.GL01099}attP2*	Bloomington	Cat# 36902; RRID: BDSC_36902
*D. melanogaster*; *UASp-Oseg6*^*shRNA*^ *y[1] sc[^∗^] v[1]; P{y[+t7.7] v[+t1.8]=TRiP.GLC01452}attP2*	Bloomington	Cat# 43263; RRID: BDSC_43263
*D. melanogaster*; *UASp-IFT52*^*shRNA*^ *y[1] v[1]; P{y[+t7.7] v[+t1.8]=TRiP.HMJ22356}attP40*	Bloomington	Cat# 58273; RRID: BDSC_58273
*D. melanogaster*; *UASp-dally*^*shRNA*^ *y[1] v[1]; P{y[+t7.7] v[+t1.8]=TRiP.JF03175}attP2*	Bloomington	Cat# 28747; RRID: BDSC_28747

**Software and Algorithms**		

Fiji	Schindelin et al., 2012	RRID: SCR 002285
GraphPad Prism 7	GraphPad Software	RRID: SCR 002798

**Deposited Data**		

RNA-seq data	ArrayExpress	E-MTAB-7063

**Other**		

Leica TCS SP8 AOBS inverted microscope	Leica	N/A

### Contact for Reagent and Resource Sharing

Further information and requests for resources and reagents should be directed to and will be fulfilled by the Lead Contact, Hilary L. Ashe (hilary.ashe@manchester.ac.uk).

### Experimental Model and Subject Details

*Drosophila* lines were maintained at 18°C while fly crosses and adult female flies for dissection were raised at 25°C, unless otherwise stated in the STAR Methods and Figure Legends, and raised on standard fly food (yeast 50g/L, glucose 78g/L, maize flour 72g/L, agar 8g/L, nipagen 27ml/L, and propionic acid 3ml/L). Adult females were raised with males for 3-7 days post-eclosion prior to dissection in order to promote normal reproductive health. The following fly lines were used in this study; *vasa.eGFP.HA* and *tkv*^*eYFP*^ (Kyoto Stock Center), *bam.GFP* (Chen and McKearin, 2003), *UASp-tkv*^*QD*^ (Tanimoto et al., 2000), *dally*^*mCh*^ (Norman et al., 2016), *BamΔ27::TIPF* (Michel et al., 2011), *dpp*^*HA*^ (Shimmi et al., 2005), and CRISPR knockin *dpp*^*mCh*^ (Fereres et al., 2018) and *tkv*^*mCh*^ (gift from T. Kornberg). *dpp*^*mCh*^ was generated by tagging Dpp after amino acid 465, as previously described by Entchev et al. (2000), and *tkv*^*mCh*^ was generated by replacing the STOP codon of endogenous *tkv* with the *mCherry* coding sequence. Both lines are homozygous viable, fertile and display no germline phenotype. The following were obtained from Bloomington Stock Centre; *dpp*^*hr92*^, *tkv*^*7*^*, Mad*^1-2^, *Med*^*13*^, *dad*^*GFP.FLAG*^*, UAS-dpp*^*GFP*^*, bab1-Gal4, tub-Gal80*^*ts*^*, nos-Gal4::VP16*, *UASp-eGFP.αTubulin84B*, *UASp-Actin42A.eGFP*, *UASp-Actin5c.mRFP*, *UASp-LifeAct.eGFP*, *UASp-FTractin.dTomato* and *UASp-shRNA* lines. Germline-specific RNAi was carried out by crossing *UASp-shRNA* fly lines to those carrying the germline driver *nos-Gal4::VP16*. For RNAi, flies were moved to 29°C for 1 week post-eclosion before dissection to enhance the knockdown. For temporal control of *dia*^*KD*^, *shi*^*KD*^ or *Gal80*^*ts*^ flies were raised at 18°C and then either kept at 18°C or moved to 25°C or 29°C for 3 days before dissection. For all drug/EGTA treatments, 3-5 day old flies were used for all samples.

### Method Details

#### RNA-seq

For each RNA sample 300-400 ovary pairs were dissected from 3-5 day old flies. Ovaries were dissected into 1x PBS on ice and incubated in 5 mg/ml collagenase IV in PBS (Worthington Biochemicals) at RT for 45 min to dissociate the tissue. Cells were washed in PBS and filtered through a 40 μm nylon mesh to remove debris before fluorescence-activated cell sorting (FACS) based on the expression of *vasa.GFP* or *bam.GFP* using a FACSAria™ Fusion cell sorter (Diva 8 Software; BD Biosciences). Cells were sorted into 1x PBS on ice and RNA was isolated using TRIzol (Invitrogen). Total RNA was processed and sequenced by the Genomic Technologies Core Facility (University of Manchester) on the Illumina Genome Analyser II. Reads were mapped to the *Drosophila* genome (dm3) and gene counts were analysed using HTSeq. DESeq2 was used to calculate differential expression between genotypes and differentially expressed genes were determined to be log2 fold change >0.5 more highly expressed than the other genotype with a p<0.05. GO term analysis was carried out using the Gene Ontology Consortium with Fisher’s Exact with FDR multiple test correction. RNA-seq data are available from ArrayExpress with the accession number E-MTAB-7063.

#### *Ex Vivo* Live Imaging

For live imaging 3-5 day old flies were dissected in Schneider’s Insect Media (Thermofisher) supplemented with 10% Fetal Bovine Serum (FBS) (Thermofisher), 1% (w/v) penicillin/streptavidin (Sigma) and individual ovarioles were separated and the overlying muscle removed to reduce movement during imaging. Ovarioles were mounted on a 35mm glass bottom tissue culture dish (World Precision Instruments) in a drop of Schneider’s Insect Media (10% FBS, 1% pen/strep) supplemented with 10 mg/ml fibrinogen (Millipore) which is spread across the glass bottom well before adding 1μl of thrombin (10 U/ml; GE Healthcare Lifesciences) to clot the fibrinogen. Schneider’s Insect Media (10% FBS, 1% pen/strep) supplemented with 200 mg/ml Human insulin (Sigma) was added and ovarioles were maintained at room temperature for 2-3 h.

Germaria were imaged using a Leica TCS SP8 AOBS inverted microscope using a HC PL APO CS2 motCORR 63x/1.2 water objective with 5-6x confocal zoom with a pinhole size of 1 AU, scan speed 400 Hz unidirectional, format 512 x 512, 2x line averaging, with 10-20 *z*-stacks taken at 0.75 μm intervals every 30-60 seconds. Imaging was carried out at room temperature for 1-3 h and images were subsequently analysed processed in Fiji, including bleach correction and a 0.5*x* x 0.5*y* x 0.5*z* pixel-wide 3D Gaussian blur. For later analysis, a brightfield image was taken to localise the GSCs and niche. False-colouring of germ cells was manually applied in Adobe Illustrator.

#### Immunofluorescence Imaging

Ovaries were fixed in 4% formaldehyde in PBT (1x PBS, 0.1% Triton X-100) for 15 min, washed three times for 15 min in PBT and blocked in 10% Bovine serum albumin (BSA) in PBT for 30 min before overnight incubation with primary antibodies in 10% BSA in PBT at 4°C. Ovaries were then washed four times in PBT over an hour and incubated with secondary antibodies for 2 h at room temperature. They were then washed twice for 15 min in PBT and once for 15 min in PBS before mounting in Prolong Gold Antifade with DAPI (Invitrogen). For phalloidin staining, ovaries were incubated for 30 min (during the second PBT wash before mounting) and washed twice for 15 min with PBT before mounting. Fixed germaria were imaged using a Leica TCS SP5 AOBS inverted microscope using an HCX PL APO 63x/1.4 oil objective or PL APO 100x/1.4 oil objective.

For visualising MT projections, flies were fixed in PEM buffer (80 mM PIPES (Sigma), 1 mM MgCl_2_ (Sigma), 5 mM EGTA (Sigma), pH 7.4) with 4% formaldehyde and 2 μM paclitaxel (Sigma) for 30 min and rinsed twice in PEM buffer and washed in 3 times for 15 min in PBT (1x PBS, 0.1% Triton X-100) before blocking in 10% BSA in PBT for 30 min. Protocol was followed as outlined above.

For visualising extracellular proteins only, ovaries were fixed in PEM buffer with 4% formaldehyde for 15 min, washed in PEM three times for 15 min and blocked for 30 mins in 5% BSA in PEM. Ovaries were incubated with primary antibodies in 5% BSA in PEM for 3 h at room temperature, washed four times in PEM over an hour before incubation with secondary antibodies for 2 h at room temperature. The ovaries were washed three times in PEM and mounted in Prolong Gold Antifade with DAPI. Antibodies were used at higher concentrations; Rt anti-DCAD2 (DSHB, 1:10), Rb anti-mCherry (ab183628, 1:50), Ms anti-HA 12CA5 (Roche, 1:50) and Rb anti-GFP (ab6556, 1:50).

To visualise endogenous TIPF and mCherry fluorescence, ovaries were fixed in PEM buffer with 4% formaldehyde for 15 min, before washing with PEM three times for 15 min. Ovaries were mounted in Prolong Gold Antifade and immediately imaged.

Antibodies used include; Rb anti-GFP (ab6556, 1:250), Goat anti-GFP (ab6673, 1:500), Rb anti-mCherry (ab183628, 1:500), Ms anti-RFP (ab65856, 1:250), Ms anti-αSpectrin 3A9 (DSHB, 1:50), Rt anti-DCAD2 (DSHB, 1:50), Ms anti-Futsch 22C10 (DSHB, 1:50) Rb anti-pSmad3 (ab52903, 1:500), Rt anti-αTubulin [YL1/2] (ab6160, 1:200), Ms anti-acetylated αTubulin [6-11b-1 (ab24610, 1:500), anti-γTubulin (Sigma GTU-88, 1:250), Rb anti-Vasa (Santa Cruz, 1:500). Secondary antibodies and other stains used were Alexa Fluor 488 Donkey anti-Rb, Alexa Fluor 488 Goat anti-Chk, Alexa Fluor 555 Donkey anti-Ms, Alexa Fluor 594, Donkey anti-Rt, Alexa Fluor 647 Donkey anti-Rt, Alexa Fluor 647 Donkey anti-Rb, Alexa Fluor 633-conjugated wheat germ agglutinin and Alex Fluor 488-conjugated Phalloidin (Thermofisher).

#### Pharmacological Inhibition

For *ex vivo* treatment with actin and MT depolymerising drugs, 5 ovary pairs per treatment were dissected in PBS and collected in Schneider’s Insect Media (10% FBS, 1% pen/strep). Ovaries were incubated with either the vehicle DMSO (Sigma), 2μM CytoD (Sigma), 10μM nocodazole (Sigma), 100μM dynasore (Sigma) or 100nm bafilomycin A1 (Sigma) for 30 mins or 90 mins at 25°C. Ovaries were rinsed with PBS and fixed. Immunostaining protocols were followed as previously described.

For EGTA treatment, 5 ovary pairs (*vasa.GFP* expressing females) per treatment were dissected in PBS and collected in Schneider’s Insect Media without FBS (1% pen/strep). Ovaries were treated with either H_2_O or 6mM EGTA for 90 or 150 mins. Ovaries were rinsed with PBS and fixed. Extracellular immunostaining protocol was followed as previously described. Endogenous Vasa.GFP expression was used for orientation when imaging.

Dextran uptake assays were performed by incubating ovaries (*shRNA* expressing or *vasa.GFP* expressing) with drugs in 100μl of media as described above for 30 mins prior to adding 3kDa Dextran-Texas Red or 10kDa Dextran-Alexa Fluor 488 (2 mg/ml; ThermoFisher) and incubating for further 60 mins. Ovaries were rinsed three times in PBS and fixed in 4% formaldehyde in PBS for 15 mins before mounting in Prolong Gold Antifade. For *shRNA* expressing germaria ovaries were also incubated with Alexa Fluor 633-conjugated wheat germ agglutinin (5 μg/ml) for 15 mins before washing and mounting in Prolong Gold Antifade.

### Quantification and Statistical Analysis

#### Quantification of Projection Length, Thickness and Dynamics

Cytocensor length was measured using the line tool in Fiji to measure from the tip of the projection to the base. Thickness was measured at the base. For actin projections dynamics, live images were maximum projected and analysed in Fiji. Length was measured using the line tool to measure from the tip of the projection to the base at the point at which it achieves its maximum length. Extension speed was measured as the time taken from the point of nucleation until the projection reaches its maximum length. Retraction speed is defined as the time it takes a projection to completely collapse after reaching its maximum length. Lifetime is measured as the total amount of time the projection is visible for. The two-dimensional angle of nucleation was measured using the angle tool in Fiji to draw a line from the centre of the niche, determined from a brightfield view of the germarium, to the centre of the stem cell or cystoblast.

#### Quantification of BMP Signaling Response and Germ Cell Number

To measure pMad fluorescence, *z*-stacks (sum of slices) were generated at 0.75μm intervals of all 10-12 slices incorporating individual GSCs. Using the draw tool in Fiji, a circle encompassing the GSC was drawn and the integrated fluorescence density (IFD) was taken and background subtracted (IFD - (average mean intensity of background x area of the region sampled)). All results were then normalised to controls. Quantification of germ cell numbers was carried out using spectrosome staining. GSCs were classed as germ cells localised at the anterior tip of the germarium in contact with the niche and containing an anteriorly anchored spectrosome. Early germ cell quantification included GSCs and any additional round spectrosome containing cells. To count TIPF puncta, a brightfield image of the germarium was used to locate the niche and TIPF puncta were manually counted on Fiji. To measure the relative ratio of pMad between GSC-pCB pairs, *z*-stacks (sum of slices) were generated at 0.75μm intervals of all 10-12 slices incorporating individual GSCs. Using the draw tool in Fiji, a circle encompassing the GSC was drawn and the IFD was used to determine the ratio of pMad fluorescence. Quantification of Rab5+ and Tkv+ endosomes in GSCs was done manually.

#### Quantification of Ecad Levels

To measure Ecad fluorescence, *z*-stacks (sum of slices) were generated at 0.75μm intervals of all 8-12 slices incorporating individual GSC-niche contact points. Using the draw tool in Fiji, a line was drawn over the area of contact between individual GSCs and neighbouring CpCs using α-Spectin staining which outlines the CpCs and GSCs. Fluorescence intensity was calculated the same as above using nuclear intensity as background. All results were then normalised to controls. n indicates individual GSC-niche contacts from n>10 germaria from at least 2 biological replicates.

#### Quantification of Dextran Uptake Assay

To determine the size of dextran^+^ vesicles, 5 *z-*slices taken of entire germaria at 1μm intervals were used and maximum projected. The resulting images were background subtracted and thresholded to generate a binary image using Fiji. The area of each dextran^+^ vesicle within the region of the germarium, including both germline and somatic cells, was then measured using analyse particles setting with a minimum size of 0.04μm^2^. The number of dextran puncta per GSC was manually counted as the total number of puncta within the anterior germarium, outlined by wheat germ agglutinin, and divided by the total number of GSCs present (2-3).

#### Statistical Analysis

Statistical comparisons were performed using two-tailed Student’s t tests, one-way ANOVA with multiple comparisons or paired t test (Figure 6C) using GraphPad Prism and Microsoft Excel. Statistical significance was assumed by p<0.05. Individual p values are indicated. Data are represented by the mean and standard deviation unless otherwise stated.

## Supplemental Information

Document S1. Figures S1–S7

Table S1. Genes Differentially Expressed between TkvQD and bamGFP-Expressing Germ Cells, Related to Figure 1

Table S2. Genes Differentially Expressed between TkvQD and bamKD-Expressing Germ Cells, Related to Figure 1

Document S2. Article plus Supplemental Information
