# Supplementary material for: Drosophila Ovarian Germline Stem Cell Cytocensor Projections Dynamically Receive and Attenuate BMP Signaling
Source: Dev Cell. 2019 Aug 5;50(3):296–312.e5. doi: 10.1016/j.devcel.2019.05.020 (PMC6688100; doi:10.1016/j.devcel.2019.05.020)
Supplement: Document S1. Figures S1–S7 [file mmc1.pdf]

Developmental Cell, Volume *50*

Supplemental Information

Drosophila Ovarian Germline Stem Cell Cytocensor Projections Dynamically Receive and Attenuate BMP Signaling

Scott G. Wilcockson and Hilary L. Ashe

Wilcockson_Figure S1

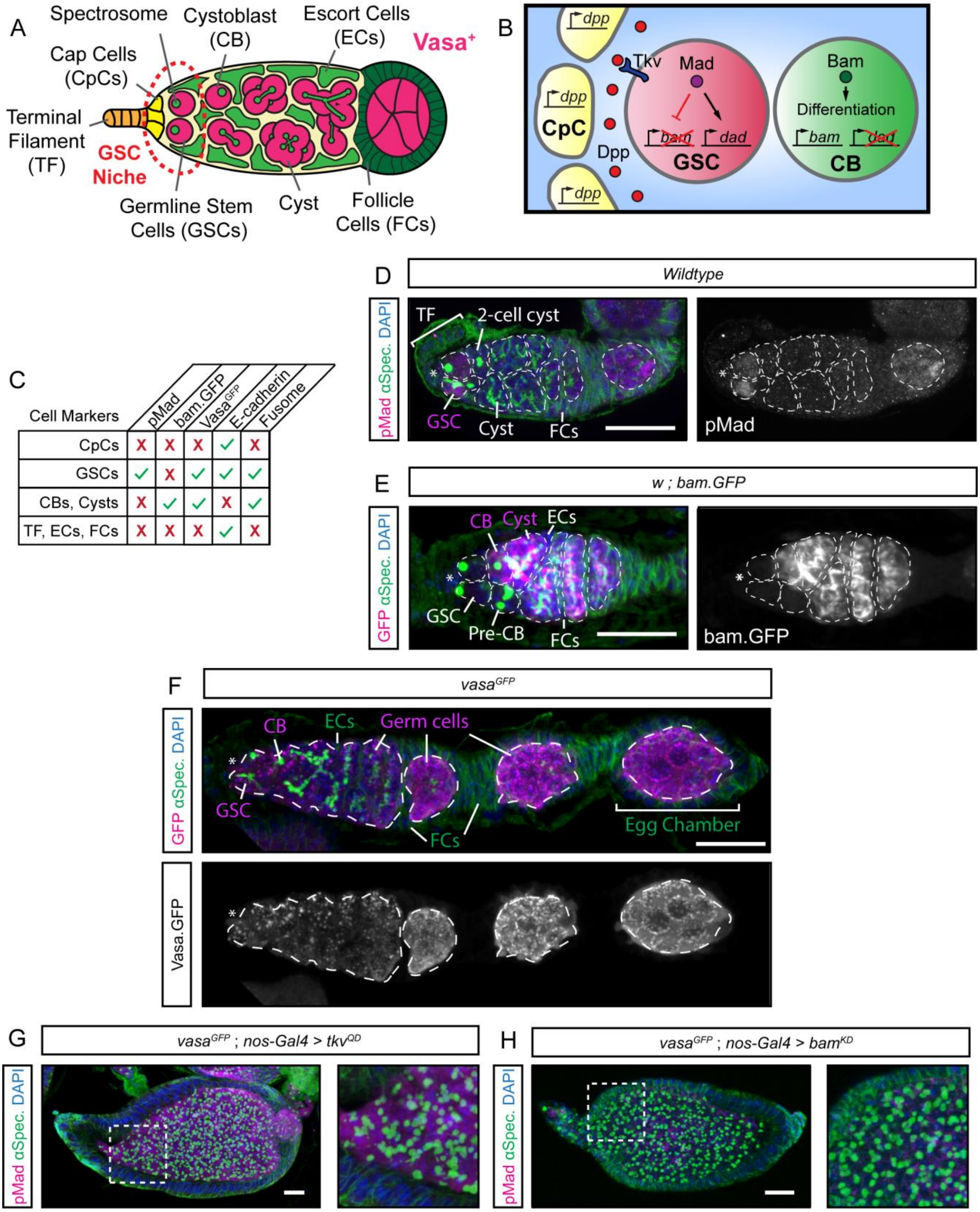

Figure S1. The *Drosophila* ovarian germline. Refers to Figure 1

(A) Cartoon depicting the structure of the germarium and GSC niche. The germline is indicated by Vasa expression (magenta). Niche cells are the somatic cells outlined by the dashed line.

(B) Cartoon depicting the regulation of GSC self-renewal and differentiation by niche Dpp signalling.

(C) Table indicating cell marker expression in all the cells of the germarium.

(D) GSCs are identifiable as anteriorly localised pMad^{+} germ cells containing a single, round spectrosome.

(E) Differentiating germ cells and cysts are identifiable by the expression of *bam* (shown here by a *bam.GFP* reporter).

(F) All germ cells are marked by the expression of *vasa* (here a *vasa.GFP* reporter).

(G) Germline-specific expression of constitutively active Tkv (Tkv^{QD}) generates tumours of pMad^{+} GSC-like cells with single, round spectrosomes. (Inset) closeup view of boxed region.

(H) Germline-specific expression of *bam*^{KD} generates tumours of pMad^{-} GSC-like cells with single, round spectrosomes. (Inset) closeup view of boxed region.

Scale bar = 5µm. CpCs (*). Dashed lines mark individual germ cells and cysts.

Wilcockson_Figure S2

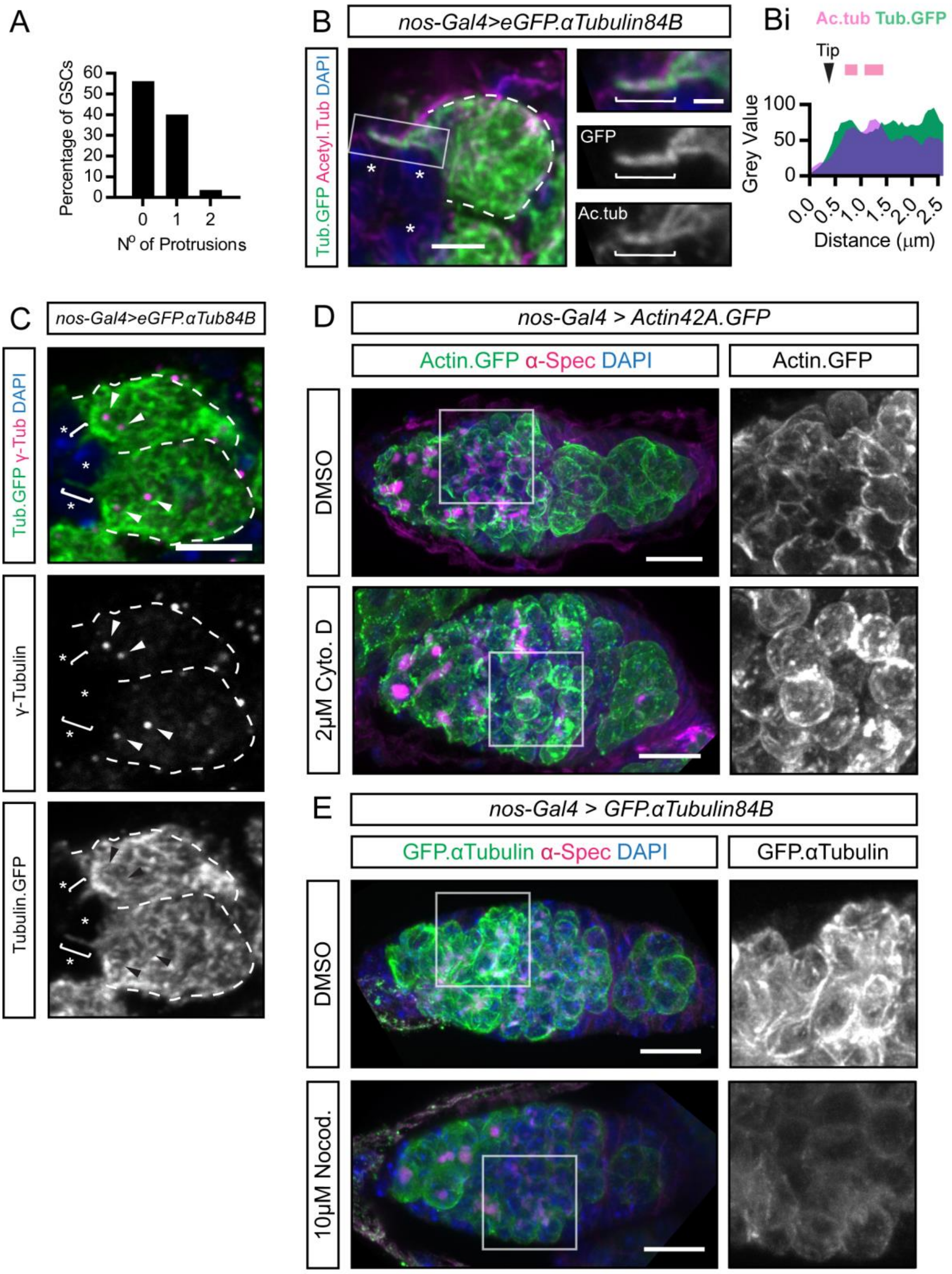

Figure S2. Composition of GSC projections. Refers to Figure 1

(A) Frequency of cytocensor formation per GSC. n=100 GSCs.

(B) GFP-αTubulin84B marked MT-projection labelling acetylated MTs. (Inset) closeup view of boxed region. (Bi) fluorescence intensity plot along the shaft of the MT-projection. Magenta lines denote highly acetylated regions.

(C) Centrosome localisation, labelled by γ-tubulin, relative to MT-projection. Brackets indicate MT-rich projections. Arrowheads indicate centrosomes. Dashed lines mark individual GSCs.

(D-E) Testing *ex vivo* drug treatment on germaria expressing Actin42A.GFP or GFP-αTubulin84B. A 30 min treatment with 2µM cytochalasin D leads to fragmentation of actin and the accumulation of puncta (D). Similar treatment with 10µM nocodazole reduces tubulin levels (E).

Scale bar = 5µm or 1µm (insets).

Wilcockson_Figure S3

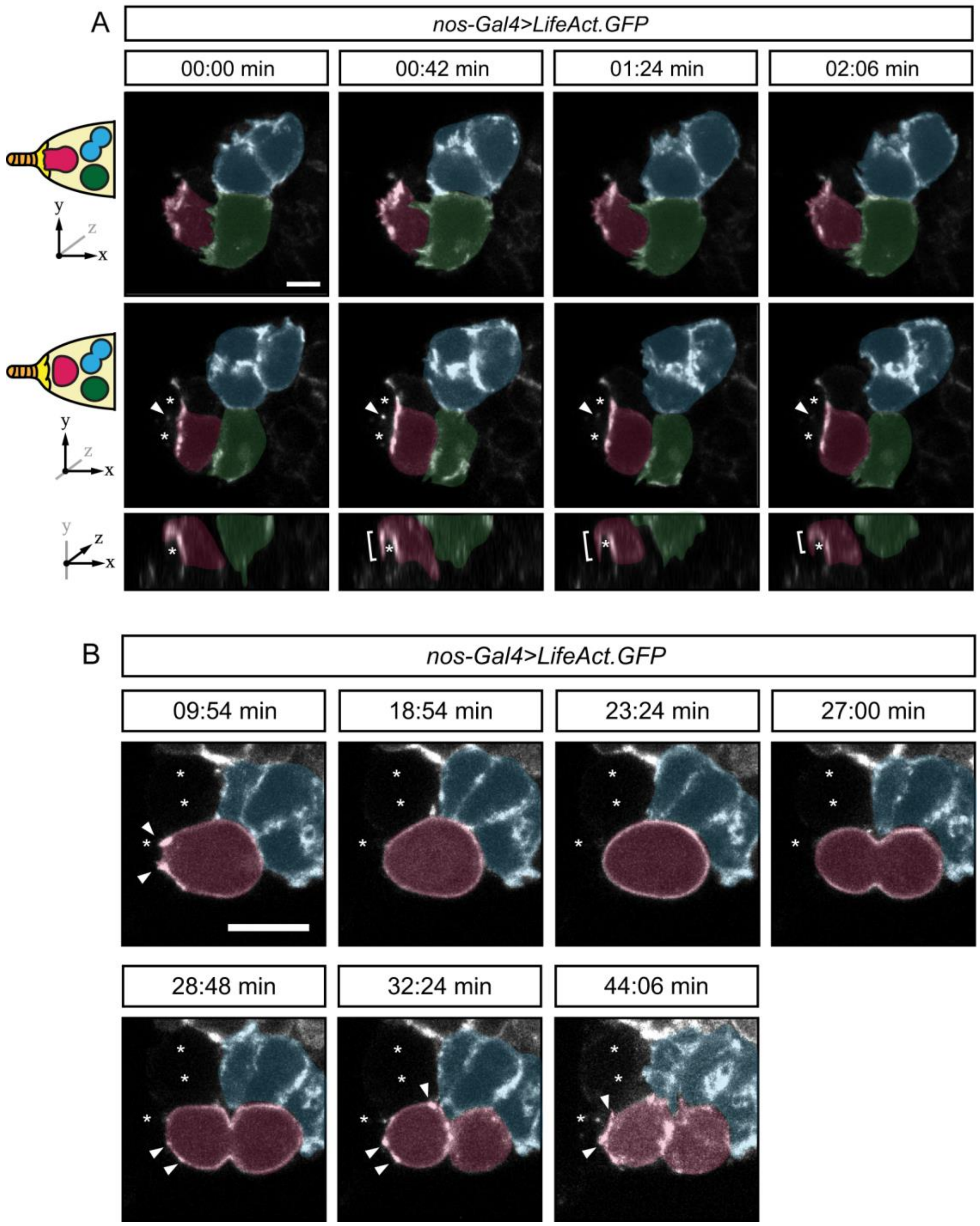

Figure S3. F-actin-rich projection dynamics. Refers to Figure 2

(A) Stills from Video 3 showing F-actin in GSCs. Top panels show first 2 slices of a maximum projection showing a broad lamellipodia-like projection depicted in cartoon form on the left and axes denote position within the maximum projection. Middle panels show 2 deeper slices in the middle revealing two CpCs (*) that the lamellipodial projections extend over. Bottom panel shows *xz*-plane view. Arrowheads mark the bisected shaft (middle) or brackets show the entire finger-like filpodium (bottom).

(B) Stills from Video 4 showing F-actin dynamics during GSC mitosis. Arrowheads indicate F-actin-rich niche-directed projections and puncta that form post-mitosis. Niche CpCs (*). Scale bar = 5μm

Wilcockson_Figure S4

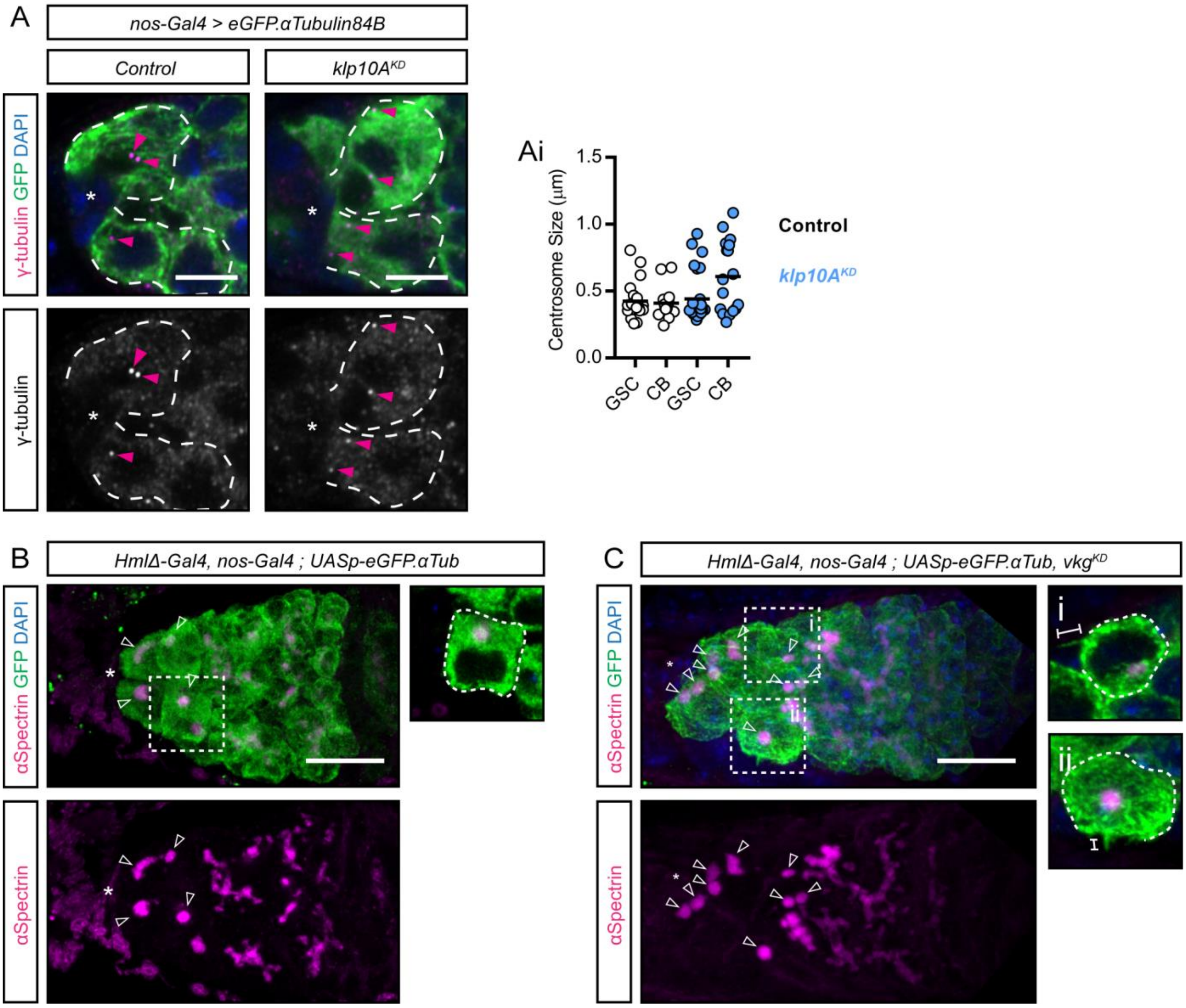

Figure S4. Ectopic Dpp signalling induces cytocensor formation. Refers to Figure 3

(A) Germline-specific *klp10A* knockdown has no effect on the GSC centrosome size (labelled with γ-tubulin, magenta arrowhead).

(Ai) Quantification for (A). Line shows mean.

(B-C) Germline and haemocyte specific expression of *eGFP-αTub84B* and RNAi-knockdown of *vkg* (ColIV) expression. (B) Control germaria have 3-4 early germ cells (open arrowheads) and cells that exit the niche (inset) do not typically generate cytocensors. (C) Knockdown of *vkg* expression in larval haemocytes extends the range of Dpp in the germarium, leading to ectopic germ cell accumulation and cytocensor formation by cells that have exited the niche (inset).

Dashed lines outline individual GSCs. (*) Niche CpCs; brackets show projections. Scale bars = 5µm (A) or 10µm (B and C).

Wilcockson_Figure S5

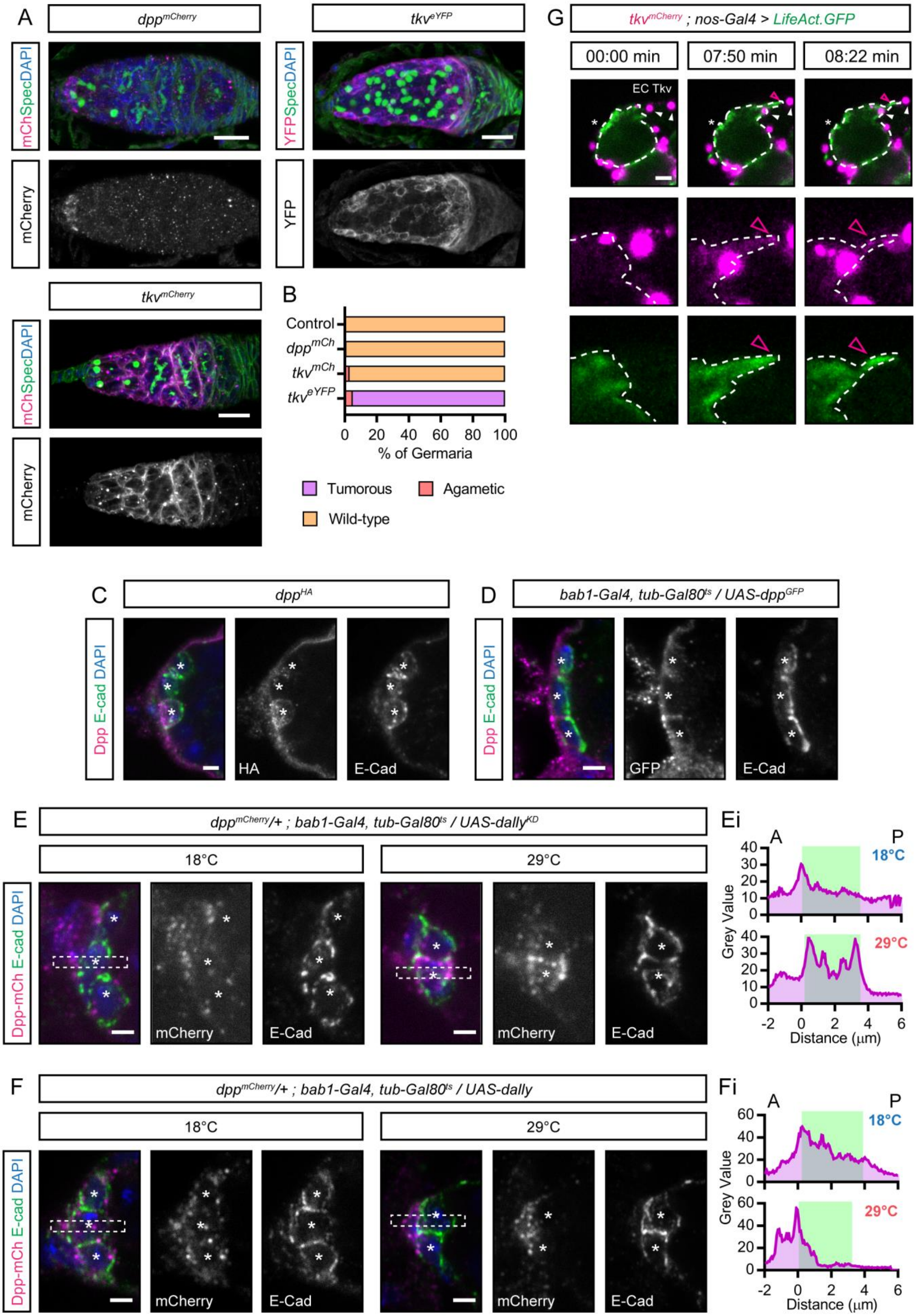

Figure S5. Niche cell-expressed Dally sequesters Dpp away from GSCs. Refers to Figures 4 and 5.

(A) Germline phenotypic analysis of *dpp*^{mCh}, *tkv*^{mCh} and *tkv*^{eYFP} lines quantified in (B) for n=100 germarium each. Control = wildtype.

(C-D) Extracellular immunostaining of tagged-Dpp transgenic lines; (C) Dpp^{HA} expressed as a transgene under the control of *dpp* regulatory elements and (D) UAS-Dpp^{GFP} which was transiently expressed in the anterior somatic cells of the germarium using *bab1-Gal4, tub-Gal80*^{ts}.

(E-F) Dpp^{mCh} localisation around the GSC niche following (E) transient knockdown or (F) overexpression of Dally in the anterior germarial somatic cells (18°C controls and 29°C *dally* knockdown/overexpression). Box shows where the plot of fluorescence intensity (Ei and Fi) was taken from anterior to posterior (A to P) through the centre of the niche (*). E-cadherin defines the niche cell boundaries (green).

(G) Stills from a video showing GSC (dashed line) F-actin labelled with *LifeAct.GFP* and endogenous mCherry-tagged Tkv showing a lateral actin-rich projection and Tkv.mCh at the tip (magenta open arrowhead). In addition, escort cell expressed 'decoy' Tkv are readily apparent in large puncta (white arrowhead) which are not connected to the GSC in the first panels.

(*) Niche CpCs; Scale bars = 2µm.

Wilcockson_Figure S6

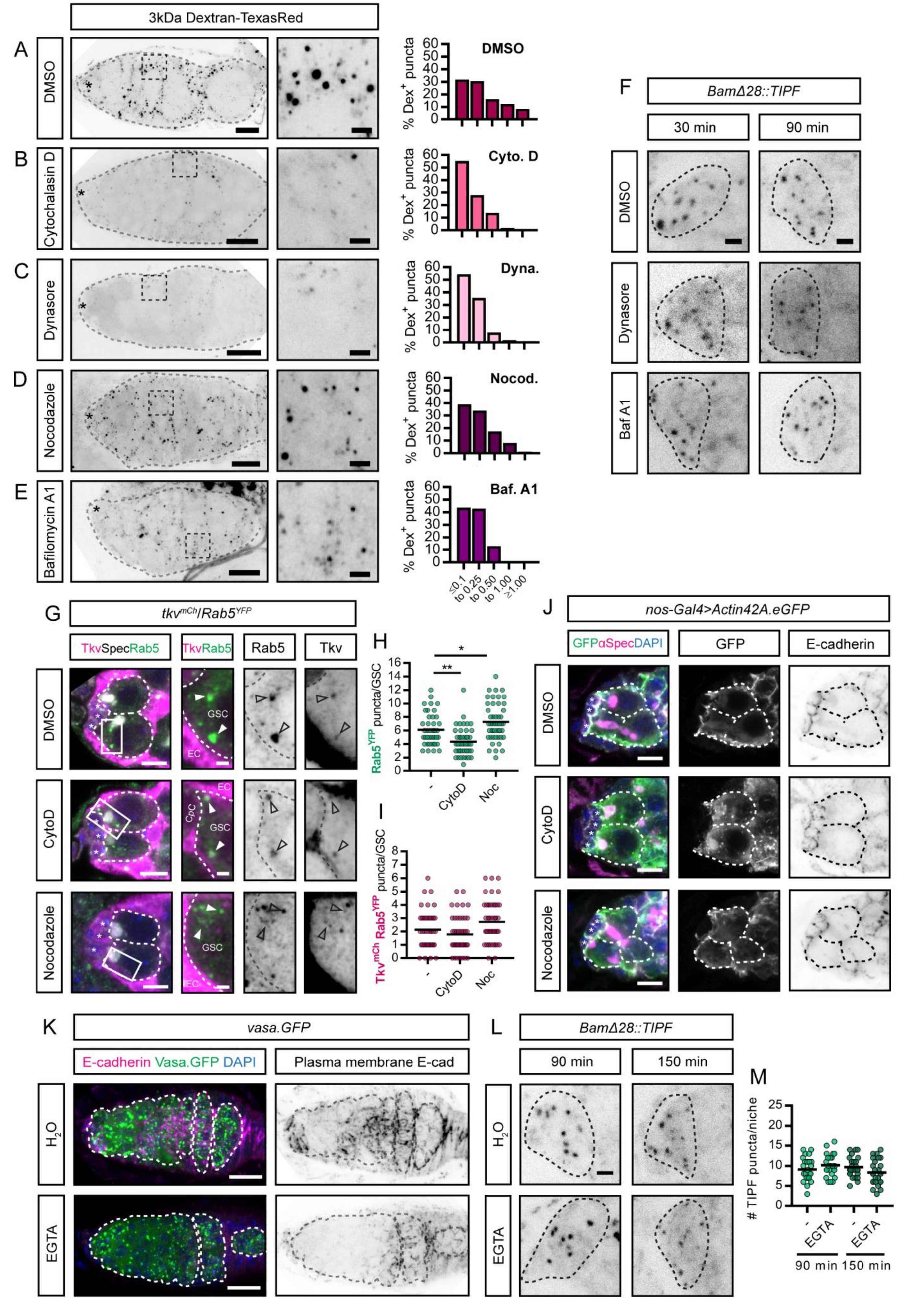

Figure S6. Short-term inhibition of Tkv endocytosis or degradation or niche adhesion does not effect Dpp signal transduction. Refers to Figure 7.

(A-E) Endogenous fluorescence of 3kDa Dextran-Texas Red in *vasa.GFP* expressing germaria (Vasa.GFP expression not shown but used for orientation when imaging). (Insets) Closeup views of the indicated boxed regions. Histograms show the Dextran labelled puncta size as a percentage of the total number of puncta for DMSO (A; n=416), CytoD (B; n=114), dynasore (C; n=112), nocodazole (D; n=241) and BafA1 (E; n=240) from 5 *z*-slices taken of the entire germarium at 1µm intervals. Grey dashed line outlines the entire germarium.

(F) Endogenous fluorescence of the TIPF reporter following 30 and 90 min treatments with DMSO (control), 100µM dynasore or 100nM BafA1 in inverted black and white for clarity. Black dashed lines outlines the niche CpCs.

(G) Immunofluorescence staining of tkv^{mCh} *and* $Rab5^{YFP}$ expressing germaria following *ex vivo* incubation with DMSO (control), 2µM CytoD or 10µM nocodazole for 90 mins before fixation. (Insets) Closeup views of the indicated boxed regions. Individual channels are in inverted black and white for clarity. Dashed lines outline GSCs. Arrowheads mark Tkv^{mCh}-positive $Rab5^{+}$ vesicles.

(H-I) Quantification of the total number of $Rab5^{+}$ vesicles per GSC (H) and the number of Tkv^{mCh}-positive $Rab5^{+}$ vesicles per GSC (I). n ≤ 44 GSCs per treatment.

(J) Immunofluorescence staining of germaria with germline-specific expression of *Actin42A.eGFP* following *ex vivo* incubation with DMSO (control), 2µM CytoD or 10µM nocodazole for 90 mins before fixation. Dashed lines outline GSCs and GSC-pCB pairs. Ecad staining is shown inverted for clarity.

(K) Extracellular staining of Ecad localisation in *vasa.GFP* expressing germaria following *ex vivo* incubation with 6mM EGTA for 90 mins. Images show antibody fluorescence for Ecad and endogenous Vasa.GFP expression. Dashed lines outline $Vasa^{+}$ germline.

(L) Endogenous fluorescence of the TIPF reporter following 90 and 150 min incubations with water (control) or 6mM EGTA in inverted black and white for clarity. Black dashed lines outline the niche CpCs.

(M) Quantification of the number of TIPF puncta per niche in (D) following water (control) or 6mM EGTA for either 90 mins (n = 25) or 150 mins (n=25 and 26, respectively).

(*) niche CpCs. Scale bar = 10µm (A-E and K), 5µm (G and J) or 1µm (F, L and insets). *, p<0.05 and **, p<0.001.

Wilcockson_Figure S7

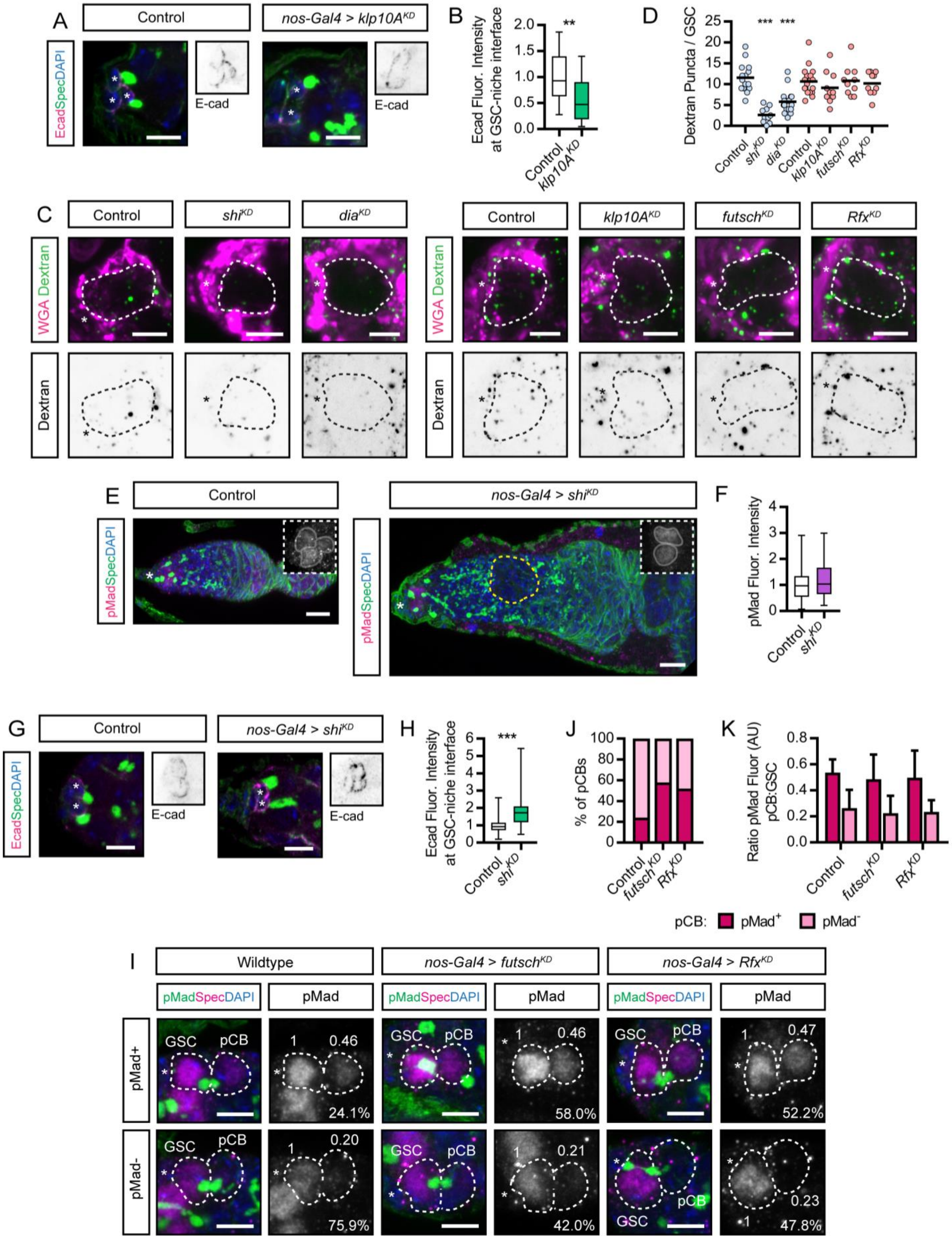

Figure S7. Altered endocytosis or pMad inheritance do not account for disrupted Dpp signal transduction following genetic manipulation of projection formation. Refers to Figure 7.

(A) Immunofluorescence staining showing germline-specific *shRNA e*xpression phenotype for control and *klp10A*^{KD} GSCs showing Ecad in inverted black and white for clarity.

(B) Quantification for (A) in control (n=31) and *klp10A*^{KD} (n=21).

(C) Endogenous fluorescence of 10kDa Dextran-Alexa Fluor 488 in GSCs expressing *shRNA* outlined by WGA-Alexa Fluor 633 and quantified in (D; n≥10). Blue indicates lines raised at 18°C and shifted to 25°C upon eclosion to induce RNAi only during adulthood. Red indicates lines raised at 25°C and shifted to 29°C upon eclosion to enhance RNAi.

(E) Immunofluorescence staining showing germline-specific *shRNA e*xpression phenotype for *shi*^{KD}*.* Early germ cells are marked by the presence of the spectrosome labelled by anti-αSpectrin. (Insets) pMad staining reports the Dpp signaling response.

(F) Quantification for (E) in control (n=53) and *shi*^{KD} (n=54).

(G) same as in (E) showing Ecad in inverted black and white for clarity.

(H) Quantification for (G) in control (n=53) and *shi*^{KD} (n=55).

(I) Immunofluorescence staining showing germline-specific *shRNA e*xpression phenotypes for *futsch* and *Rfx*. pMad staining reports the Dpp signalling response in GSC-pCB pairs (dashed line) identified by the shared spectrosome labelled by anti-αSpectrin. Numbers indicate the pMad levels relative to the indicated GSC and the percentage of GSC-pCB pairs that possess a pMad^{+} or pMad^{-} pCB.

(J-K) Quantification for (I) showing (J) the percentage of GSC-pCB pairs that possess a pMad^{+} or pMad^{-} pCB and (K) the average pMad fluorescence ratio between the GSC-pCB within the pMad^{+} or pMad^{-} groups as indicated in (J).

(*) niche CpCs. Scale bar = 10µm (E) and 5µm (A, C, G and I). For box and whisker graphs the box shows median, 25th and 75th percentile and whiskers show minima and maxima. **, $p<0.001$; ***, $p<0.0001$.
